# Cross-points in the Dirichlet-Neumann method I: well-posedness and convergence issues

**DOI:** 10.1007/s11075-022-01445-1

**Published:** 2022-11-08

**Authors:** Bastien Chaudet-Dumas, Martin J. Gander

**Affiliations:** Section de Mathématiques, Université de Genève, Geneva, CH-1205 Switzerland

**Keywords:** Domain decomposition methods, Dirichlet-Neumann methods, Cross-points, Elliptic problem, 35J05, 35D35, 65N55, 65N06

## Abstract

Cross-points in domain decomposition, i.e., points where more than two subdomains meet, have received substantial attention over the past years, since domain decomposition methods often need special attention in their definition at cross-points, in particular if the transmission conditions of the domain decomposition method contain derivatives, like in the Dirichlet-Neumann method. We study here for the first time the convergence of the Dirichlet-Neumann method at the continuous level in the presence of cross-points. We show that its iterates can be uniquely decomposed into two parts, an even symmetric part that converges geometrically, like when there are no cross-points present, and an odd symmetric part, which generates a singularity at the cross-point and is not convergent. We illustrate our analysis with numerical experiments.

## Introduction

Domain decomposition methods for partial differential equations are naturally defined and analyzed at the continuous level, like the original overlapping Schwarz method from 1869 [1], and also the original Dirichlet-Neumann [2] and the Neumann-Neumann [3] method. Even FETI (Finite Element Tearing and Interconnect) was first presented at the continuous level in the original publication [4], as a minimization problem, before the authors proceeded to the finite element discretization that led to its name. For symmetric and positive definite problems, early domain decomposition research focused then however on condition number estimates for such methods used as preconditioners at the discrete level, leading to groundbreaking results (see, e.g., [5] and references therein). The methods were thus intimately linked with the conjugate gradient method and not considered as standalone solvers, in contrast to multigrid methods, for example [6, 7]. For more general problems which are non-symmetric and/or indefinite, condition numbers are not the key quantities anymore for understanding their convergence when used as preconditioners, and directly studying preconditioning properties for more general Krylov methods becomes difficult. There has therefore been an effort to also investigate the underlying iterative versions of these methods, and the study of their convergence properties at the continuous level (see, for example, [8] and references therein). This reveals many interesting properties of domain decomposition methods which are masked by the Krylov method that can correct convergence problems when the domain decomposition method is used as preconditioner. An interesting example is the Additive Schwarz method, which needs Krylov acceleration to be used, while Restricted Additive Schwarz does not, since it corresponds directly to the discretization of the parallel Schwarz method of Lions [9], and thus converges as a standalone iterative method [10, 11].

We are interested here in understanding the convergence properties of the Dirichlet-Neumann method in the presence of cross-points. Cross-points in domain decomposition methods have become a focus of attention over the past years because of an increasing interest in the domain decomposition research community to better understand the discretization of domain decomposition methods at cross-points, and the influence on the convergence of the iterative solution. For the Helmholtz equation and Després’ seminal non-overlapping Schwarz method with Robin transmission conditions, cross-points do not hamper convergence [12], and the same holds more generally for non-overlapping optimized Schwarz methods when studied at the continuous level (see [13]). Care needs to be taken however when discretizing such methods (see, for example, [14–18]), and this is even more important when higher order transmission conditions like Ventcell conditions are used [19–23]. For the Neumann-Neumann method, a well-posedness issue has been identified in the presence of cross-points (see [24]), and the authors present a modification of the method to get around this difficulty. Also multitrace formulations pose problems at cross-points, and a solution involving specific non-local operators has been proposed in [25] (see also [26] for a purely algebraic formulation). There was even a dedicated mini-symposium on cross-points in domain decomposition methods at the last international conference on domain decomposition methods, DD27, in Hong Kong [27].

For the Dirichlet-Neumann method, the well-posedness issue in the presence of cross points was already mentioned in early work [28], but has so far not been fully analyzed for the iterative version of the algorithm. Most research works using this method or variants of this method do not encounter this problem. Indeed, many authors consider the case of domain decompositions with two subdomains [2, 29, 30] or many subdomains in stripes [31, 32], which excludes the presence of cross-points. Others use the Dirichlet-Neumann method as a preconditioner for a Krylov method (see, for example, [8, 33, 34]), which hides the problematic behavior of the Dirichlet-Neumann method. We focus here on a simple but instructive Laplace problem on a square divided into four squared subdomains of equal area. This allows us to develop a complete analytical understanding of the convergence of the Dirichlet-Neumann method in the presence of cross-points at the continuous level. We will show that the even symmetric part of the iteration converges like when no cross-points are present, but the odd symmetric part of the iteration generates singularities at cross-points and is thus not convergent. Even though our analysis is limited to the case of four subdomains, it reveals the behavior of the iterates locally at the cross-point. Therefore, it is representative of the behavior of the iterates near cross-points for more general domain decompositions of grid-shape. The study on how to correct the convergence problem of the odd symmetric part will be addressed in a further research paper.

## Geometry and model problem

**Fig. 1:**
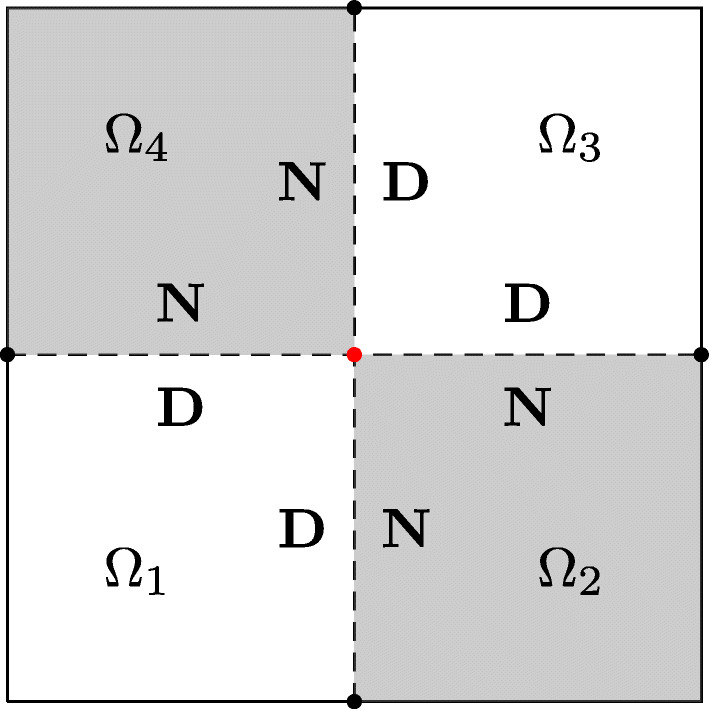
Domain Ω divided into four square subdomains colored in gray and white

As shown in Fig. 1, we use as domain ${\Omega }\subset \mathbb {R}^{2}$ for our Laplace model problem the square (− 1,1) × (− 1,1), divided into four non-overlapping square subdomains Ω_*i*_, $i\in \mathcal {I}:=\{1,2,3,4\}$, of equal area. With such a partition, there is one interior cross-point (red dot), and there are also four boundary cross-points (black dots). The presence of boundary cross-points has been identified as an obstacle for multitrace formulations, which requires some additional specific treatment (see, for example, [35, Section 2.4]). Conversely, these points are not an issue here for the Dirichlet-Neumann method. We denote the interfaces between adjacent subdomains by Γ_*ij*_ := int(*∂*Ω_*i*_ ∩ *∂*Ω_*j*_), and the skeleton of the partition by ${\Gamma }:=\bigcup _{i,j} \overline {\Gamma }_{ij}$, and we have Γ_*ij*_ = Γ_*ji*_ for all *i*, *j*. We further denote the interior of the intersection between *∂*Ω_*i*_ and the boundary *∂*Ω by ${\partial {\Omega }_{i}^{0}}:=\text {int}(\partial {\Omega }_{i}\cap \partial {\Omega })$, and the interior of the left, right, bottom, and top sides of Ω by *∂*Ω_*l*_, *∂*Ω_*r*_, *∂*Ω_*b*_, and *∂*Ω_*t*_. Thus, an arbitrary side of Ω is denoted by *∂*Ω_*σ*_, where *σ* is in the set of indices $\mathcal {S}:=\{l,r,b,t\}$. We use the same notation also for an arbitrary side of a subdomain Ω_*i*_, namely *∂*Ω_*i*,*σ*_ with $\sigma \in \mathcal {S}$.

### Even and odd symmetric functions

We recall now the definition of an even/odd symmetric function in multivariate calculus, and a useful decomposition result, which we express here in terms of functions in *L*^*p*^, for $p\in [1,+\infty ]$.

#### **Definition 1** (Symmetric set)

Let *n* ≥ 1 be an integer. A subset *U* of $\mathbb {R}^{n}$ is said to be *symmetric* if, for any $(x_{1}, \cdots ,x_{n})\in \mathbb {R}^{n}$,

$$ (x_{1}, \cdots,x_{n})\in U  \Longrightarrow  (-x_{1}, \cdots,-x_{n})\in U . $$

#### **Definition 2** (Even/odd symmetric functions)

Let $U\subset \mathbb {R}^{n}$ be open and symmetric, and $p\in [1,+\infty ]$. A function *h* ∈ *L*^*p*^(*U*) is called *even symmetric* if for almost all (*x*_1_,⋯ ,*x*_*n*_) ∈ *U*,

$$ h(-x_{1}, \cdots,-x_{n}) = h(x_{1}, \cdots,x_{n}) . $$

Similarly, the function *h* is called *odd symmetric* if for almost all (*x*_1_,⋯ ,*x*_*n*_) ∈ *U*,

$$ h(-x_{1}, \cdots,-x_{n}) = -h(x_{1}, \cdots,x_{n}) . $$

#### **Theorem 1** (Even/odd decomposition)

Let $U\subset \mathbb {R}^{n}$ be open and symmetric, and $p\in [1,+\infty ]$. Every function *h* ∈ *L*^*p*^(*U*) can be uniquely decomposed as the sum of an even and an odd symmetric function, both in *L*^*p*^(*U*), which are called the *even symmetric part* and the *odd symmetric part* of the function. These functions, denoted by *h*_*e*_ and *h*_*o*_, are for almost all (*x*_1_,⋯ ,*x*_*n*_) ∈ *U* given by

$$ \begin{array}{@{}rcl@{}} h_{e}(x_{1}, \cdots,x_{n}) &:=& \frac{1}{2}\left( h(x_{1}, \cdots,x_{n}) + h(-x_{1}, \cdots,-x_{n}) \right) , \\ h_{o}(x_{1}, \cdots,x_{n}) &:=& \frac{1}{2}\left( h(x_{1}, \cdots,x_{n}) - h(-x_{1}, \cdots,-x_{n}) \right) . \end{array} $$

#### *Proof 1*

Taking the sum, we see that *h*_*e*_ + *h*_*o*_ = *h* almost everywhere (a.e.) in *U*. Then, regarding uniqueness, let us assume that there exists another couple of even/odd symmetric functions $(\tilde {h}_{e},\tilde {h}_{o})\neq (h_{e},h_{o})$ such that $\tilde {h}_{e}+\tilde {h}_{o}=h$ a.e. in *U*. It follows that

$$ (h_{e}+h_{o})(x_{1}, \cdots,x_{n}) = (\tilde{h}_{e}+\tilde{h}_{o})(x_{1}, \cdots,x_{n}) ,  \text{ for almost all } (x_{1}, \cdots,x_{n}) \in U . $$

This implies that $h_{e}-\tilde {h}_{e}=\tilde {h}_{o}-h_{o}$ a.e. in *U*. Since the left hand-side is even symmetric and the right hand-side is odd symmetric, we must have $h_{e}-\tilde {h}_{e}=\tilde {h}_{o}-h_{o}=0$ a.e. in *U*, which contradicts the initial assumption. □

#### **Definition 3** (Symmetry preservation of operators)

Let X and Y be two Banach spaces such that *X* ⊂ *L*^*p*^(*U*) and *Y* ⊂ *L*^*q*^(*V* ) for some open symmetric sets *U*, *V* and some *p*, $q\in [1,+\infty ]$. An operator *T* : *X* → *Y* is said to *preserve symmetry* if it satisfies the following properties:

- for all *h* ∈ *X* such that *h* is even symmetric, *Th* is even symmetric,
- for all *h* ∈ *X* such that *h* is odd symmetric, *Th* is odd symmetric.

The Laplace operator Δ : *H*^2^(Ω) → *L*^2^(Ω) preserves symmetry, which can be proved using the standard chain rule for *C*^2^ functions together with the density of $C^{\infty }(\overline {\Omega })$ in *H*^2^(Ω). In the same way, if *p* denotes an even symmetric function in $W^{\frac {1}{2},\infty }(\partial {\Omega })$, one can prove that the operator $(\partial _{n}+p):H^{\frac {3}{2}}(\partial {\Omega })\to H^{\frac {1}{2}}(\partial {\Omega })$ also preserves symmetry because for each *x* ∈Γ, *n*(−*x*) = −*n*(*x*).

### Laplace model problem

We consider the Laplace problem with Dirichlet boundary condition on Ω, that is: find *u* solution to

1 $$ \left\{ \begin{aligned} -{\Delta} u &= f  \text{ in } {\Omega} , \\ u &= g \text{ on } \partial{\Omega} , \end{aligned} \right.  $$

where *f* ∈ *H*^− 1^(Ω) and $g\in H^{\frac {1}{2}}(\partial {\Omega })$. Of course, since Ω is Lipschitz, it is known that (1) admits a unique solution *u* ∈ *H*^1^(Ω). We also consider the Laplace problem with Robin boundary condition: find *u* solution to

2 $$ \left\{ \begin{aligned} -{\Delta} u &= f  \text{ in } {\Omega} , \\ (\partial_{n}+p)u &= g \text{ on } \partial{\Omega} , \end{aligned} \right.  $$

where $p\in L^{\infty }(\partial {\Omega })$ (*p* ≥ 0 a.e. on *∂*Ω) is even symmetric, *f* ∈ *H*^− 1^(Ω) and $g\in H^{-\frac {1}{2}}(\partial {\Omega })$. To ensure that the problem is well-posed in *H*^1^(Ω), we assume that *p* is strictly positive on a subset of *∂*Ω of non-zero measure.

### Regularity results

In this section, we briefly recall some important results about the theory of elliptic boundary value problems in nonsmooth two-dimensional domains, adapted to our specific context of a square. The general results in arbitrary polygons, together with their proofs, can be found in [36, Chapter 4], [37], and [38]. For a brief review on the subject, the reader is also referred to the lecture notes [39, Chapter 2].

#### *H*^1^(Ω) regularity

As it has just been mentioned, the standard variational approach to study problems (1) and (2) leads to existence and uniqueness of solutions in *H*^1^(Ω). However, for this result to hold, the regularity required on the boundary data is $g\in H^{\frac {1}{2}}(\partial {\Omega })$ for the Dirichlet case and $g\in H^{-\frac {1}{2}}(\partial {\Omega })$ for the Neumann (Robin) case. This means that *g* can be identified as the trace on *∂*Ω of a function in *H*^1^(Ω) in the Dirichlet case. Similarly, in the Neumann (Robin) case, it can be identified as the normal derivative on *∂*Ω of a function in *H*^1^(Ω). When Ω is a polygon, the regularity on *∂*Ω can be expressed by means of the regularity on each side of the polygon associated with so-called *compatibility relations* at the corners (see [37, Chapter 1]). Let us introduce the set of corners $\mathcal {C}$ which consists in four pairs of indices in $\mathcal {S}$:

$$ \mathcal{C} := \left\{ (l,b), (r,b), (r,t), (l,t) \right\} . $$

For the reader’s convenience, the restriction of *g* to a part of the boundary *∂*Ω_*σ*_ will be denoted by $g_{\sigma }:=g\mid _{\partial {\Omega }_{\sigma }}$, for each $\sigma \in \mathcal {S}$. With these notations, we have the following useful characterization for the Dirichlet case: $g\in H^{\frac {1}{2}}(\partial {\Omega })$ *iff*

3 $$ g_{\sigma}\in H^{\frac{1}{2}}(\partial{\Omega}_{\sigma}),  \forall \sigma\in \mathcal{S}, \text{ and }  {\int}_{0}^{\varepsilon} \big\lvert g_{\sigma}(s) - g_{\sigma^{\prime}}(-s) \big\rvert^{2} \frac{\mathrm{d}s}{s} < +\infty,  \forall (\sigma,\sigma^{\prime})\in\mathcal{C},  $$

for some small *ε* > 0. There exists a similar (and simpler) characterization for the Robin case: $g\in H^{-\frac {1}{2}}(\partial {\Omega })$ *iff*

4 $$ g_{\sigma}\in H^{-\frac{1}{2}}(\partial{\Omega}_{\sigma}),  \forall \sigma\in \mathcal{S}.  $$

Therefore, replacing the regularity conditions on the boundary data *g* in problems (1) and (2) by conditions (3) and (4) leads to well-posed formulations in *H*^1^(Ω).

#### *H*^2^(Ω) regularity

The existence and uniqueness results mentioned above only give us *H*^1^(Ω) regularity for the solution, which means *u* might not even be continuous since ${\Omega }\subset \mathbb {R}^{2}$. Since we are interested in pointwise properties, especially what happens at the cross-point, we need more regular functions.

#### **Theorem 2**

(Dirichlet case) If in addition to the previous assumptions, we have *f* ∈ *L*^2^(Ω), $g_{\sigma }\in H^{\frac {3}{2}}(\partial {\Omega }_{\sigma })$ for all $\sigma \in \mathcal {S}$, and $g_{\sigma }(0)=g_{\sigma ^{\prime }}(0)$ for all $(\sigma ,\sigma ^{\prime })\in \mathcal {C}$, then the solution *u* to (1) is in *H*^2^(Ω).

#### **Theorem 3**

(Robin case) If in addition to the previous assumptions, we have *f* ∈ *L*^2^(Ω), $p\mid _{\partial {\Omega }_{\sigma }}\in C^{\infty }(\overline {\partial {\Omega }}_{\sigma })$, and $g_{\sigma }\in H^{\frac {1}{2}}(\partial {\Omega }_{\sigma })$ for all $\sigma \in \mathcal {S}$, then the solution *u* to (2) is in *H*^2^(Ω).

We will also need regularity results for the mixed (Dirichlet/Neumann) problem for subproblems generated by the Dirichlet-Neumann method: let $\mathcal {D}$ and $\mathcal {N}$ be two subsets of $\mathcal {S}$ corresponding to the indices for Dirichlet and Neumann boundary conditions. In order to avoid well-posedness issues, we assume that $\mathcal {D}\neq \emptyset $. The mixed problem reads: find *u* solution to

5 $$ \left\{ \begin{aligned} -{\Delta} u &= f  \text{ in } {\Omega} , \\ u &= g^{\mathcal{D}}_{\sigma} \text{ on } \partial{\Omega}_{\sigma}, \text{ for } \sigma \in \mathcal{D} , \\ \partial_{n} u &= g^{\mathcal{N}}_{\sigma} \text{ on } \partial{\Omega}_{\sigma}, \text{ for } \sigma \in \mathcal{N} . \end{aligned} \right.  $$

The set of corners $\mathcal {C}$ can be split into three subsets $\mathcal {C}=\mathcal {C}_{\mathcal {D}}\cup \mathcal {C}_{\mathcal {N}}\cup \mathcal {C}_{{\mathscr{M}}}$ corresponding to Dirichlet corners, Neumann corners, or mixed corners, such that for each $(\sigma ,\sigma ^{\prime })\in \mathcal {C}$,

$$ (\sigma,\sigma^{\prime}) \in \left\{ \begin{aligned} \mathcal{C}_{\mathcal{D}}  & \text{ if } \sigma,\sigma^{\prime} \in \mathcal{D}, \\ \mathcal{C}_{\mathcal{N}}  & \text{ if } \sigma,\sigma^{\prime} \in \mathcal{N}, \\ \mathcal{C}_{\mathcal{M}}  & \text{ if } \sigma \in \mathcal{D}, \sigma^{\prime} \in \mathcal{N}. \end{aligned} \right. $$

#### **Theorem 4**

(Dirichlet/Neumann case) If in addition to the previous assumptions, we have *f* ∈ *L*^2^(Ω), $g^{\mathcal {D}}_{\sigma }\in H^{\frac {3}{2}}(\partial {\Omega }_{\sigma })$ for $\sigma \in \mathcal {D}$, $g^{\mathcal {N}}_{\sigma }\in H^{\frac {1}{2}}(\partial {\Omega }_{\sigma })$ for $\sigma \in \mathcal {N}$, and

$$ \begin{aligned} g^{\mathcal{D}}_{\sigma}(0)=g^{\mathcal{D}}_{\sigma^{\prime}}(0) , \quad & \forall (\sigma,\sigma^{\prime})\in \mathcal{C}_{\mathcal{D}} ,\\ {\int}_{0}^{\varepsilon} \big\lvert {g^{\mathcal{D}}_{\sigma}}^{\prime}(s) - g^{\mathcal{N}}_{\sigma^{\prime}}(-s) \big\rvert^{2} \frac{\mathrm{d}s}{s} < +\infty , \quad & \forall (\sigma,\sigma^{\prime})\in\mathcal{C}_{\mathcal{M}}, \end{aligned} $$

then the solution *u* to (5) is in *H*^2^(Ω).

Finally, let us state one last useful result about the regularity of such functions, which is a direct consequence of the Sobolev embedding theorem (see, for example, [40]).

#### **Proposition 5**

Let *U* be a bounded Lipschitz open subset of $\mathbb {R}^{2}$. Then, any function in *H*^2^(*U*) is also in $C^{0}(\overline {U})$.

#### *Proof 2*

From the Sobolev embedding theorem, since $2>\frac {3}{2}$ and $U\subset \mathbb {R}^{2}$, we know that *H*^2^(*U*) is (continuously) embedded in the Hölder space $C^{0,\mu }_{b}(U)$ for some *μ* ∈ (0,1). In addition, any function in $C^{0,\mu }_{b}(U)$ is uniformly continuous on *U*; therefore, it can be extended to a continuous function on $\overline {U}$. □

From now on, we will assume that the data satisfy the regularity required by the assumptions in Theorems 2 and 3. This ensures that the regularity of the solutions we deal with is at least *H*^2^(Ω).

### Even/odd symmetric decomposition

We now decompose the problems (1) and (2) into two subproblems, in order to analyze the subproblems separately. With Theorem 1, we can uniquely decompose the data, and thus the associated problem, into the *even symmetric part* and the *odd symmetric part*. For problem (1), this leads to: find *u*^*e*^ and *u*^*o*^ solutions to

6 $$ \left\{ \begin{aligned} -{\Delta} u^{e} &= f_{e}  \text{ in } {\Omega} , \\ u^{e} &= g_{e} \text{ on } \partial{\Omega} , \end{aligned} \right.  $$

7 $$ \left\{ \begin{aligned} -{\Delta} u^{o} &= f_{o}  \text{ in } {\Omega} , \\ u^{o} &= g_{o} \text{ on } \partial{\Omega} . \end{aligned} \right.  $$

These subproblems are still well-posed and the solutions still have *H*^2^(Ω) regularity. Using the symmetry preserving property of the operator Δ : *H*^2^(Ω) → *L*^2^(Ω), we see that *u*^*e*^ is even symmetric and *u*^*o*^ is odd symmetric. Moreover, since (−Δ) is linear and the boundary conditions are linear as well, *u*^*e*^ + *u*^*o*^ solves (1). Therefore, *u*^*e*^ + *u*^*o*^ = *u*, and since the decomposition is unique, the solution to (6) *u*^*e*^ coincides with *u*_*e*_, the even symmetric part of *u*, and similarly *u*^*o*^ = *u*_*o*_. As we will see, the convergence analysis of the Dirichlet-Neumann method reveals different behaviors for the even and odd symmetric subproblems. Note that the geometric domain decomposition itself is also symmetric with respect to the origin (0,0).

As for the Dirichlet problem, we define the even and odd symmetric parts of (2), and still denote by *u*^*e*^ and *u*^*o*^ their solutions. When *p* is even symmetric, in addition to the operator Δ, we have seen that the operator $(\partial _{n}+p):H^{\frac {3}{2}}(\partial {\Omega })\to H^{\frac {1}{2}}(\partial {\Omega })$ also preserves symmetry. Hence, the even and odd symmetry properties of the solutions *u*^*e*^ and *u*^*o*^. Thus, the linearity of (2) yields *u*^*e*^ + *u*^*o*^ = *u*, and the uniqueness of the decomposition finally leads to *u*^*e*^ = *u*_*e*_ and *u*^*o*^ = *u*_*o*_ like in the Dirichlet case.

## Analysis of the Dirichlet-Neumann method

We use as in [8, Section 1.4] a gray and white coloring (see Fig. 1) and define the sets of indices $\mathcal {I}_{G}:=\{1\leq i\leq 4 : {\Omega }_{i} \text { is gray }\} = \{2,4\}$ and $\mathcal {I}_{W}:=\mathcal {I}\setminus \mathcal {I}_{G} = \{1,3\}$. The transmission conditions are indicated in Fig. 1, where “*D*” stands for Dirichlet and “*N*” for Neumann. Given an initial guess *u*^0^ and a relaxation parameter $\theta \in \mathbb {R}$, each iteration *k* ≥ 1 of the method can be split into two steps:

- **(Dirichlet step)** Solve for all $i\in \mathcal {I}_{W}$
  $$ \left\{ \begin{aligned} -{\Delta} {u_{i}^{k}} &= f  \text{ in } {\Omega}_{i} , \\ {u_{i}^{k}} &= g  \text{ on } {\partial{\Omega}_{i}^{0}} , \\ {u_{i}^{k}} &= \theta u_{j}^{k-1} + (1-\theta)u_{i}^{k-1} \text{ on } {\Gamma}_{ij},  \forall j\in \mathcal{I}_{G}  \text{ s.t. } {\Gamma}_{ij}\neq\emptyset . \end{aligned} \right. $$
- **(Neumann step)** Solve for all $i\in \mathcal {I}_{G}$
  $$ \left\{ \begin{aligned} -{\Delta} {u_{i}^{k}} &= f  \text{ in } {\Omega}_{i} , \\ {u_{i}^{k}} &= g  \text{ on } {\partial{\Omega}_{i}^{0}} , \\ \partial_{n_{i}}{u_{i}^{k}} &= -\partial_{n_{j}}{u_{j}^{k}} \text{ on } {\Gamma}_{ij},  \forall j\in \mathcal{I}_{W}  \text{ s.t. } {\Gamma}_{ij}\neq\emptyset . \end{aligned} \right. $$

To start the Dirichlet-Neumann method, we need an initial guess *u*^0^, or equivalently *λ*^0^ := *u*^0^∣_Γ_, since only the traces of *u*^0^ on the interfaces Γ_*ij*_ are used in the initialization step *k* = 1. This initial guess needs to satisfy the following compatibility condition.

### **Definition 4** (Compatible initial guess)

An initial guess *u*^0^ (or equivalently *λ*^0^) is said to be compatible with the Dirichlet boundary condition if it satisfies *u*^0^∣_*∂*Ω∩Γ_ = *g*∣_Γ_ (or equivalently *λ*^0^ = *g* a.e. on *∂*Ω ∩Γ representing the set of boundary cross-points).

In what follows, we fix an initial guess *λ*^0^ such that $\lambda ^{0} \in C^{0}({\Gamma })\cap H^{\frac {3}{2}}({\Gamma }_{ij})$ for all (*i*,*j*) such that Γ_*ij*_≠*∅*, and *λ*^0^ is compatible with the boundary condition.

### Case of the even symmetric part

We begin with applying the Dirichlet-Neumann method to the even symmetric part of problem (1).

#### **Theorem 6**

Taking ${\lambda ^{0}_{e}}$ as the initial guess for the Dirichlet-Neumann method applied to the even symmetric part of problem (1) produces a sequence $\{{u_{e}^{k}}\}_{k}$ that converges geometrically to the solution *u*_*e*_ in the *L*^2^-norm and the broken *H*^1^-norm, for any *𝜃* ∈ (0,1). Moreover, the convergence factor is given by ∣1 − 2*𝜃*∣, which also proves that this method becomes a direct solver for the specific choice $\theta =\frac {1}{2}$.^1^

#### *Proof 3*

We perform the first two iterations of the Dirichlet-Neumann method in terms of the local errors ${e_{i}^{k}}:=u_{i}-{u_{i}^{k}}$ where $u_{i}:=u\mid _{{\Omega }_{i}}$ is the restriction of the original solution to the *i*–th subdomain (see Section 4 (Example 1) for a numerical illustration).

**Iteration** ***k = 1***, Dirichlet step: In Ω_1_, we solve

$$ \left\{\begin{aligned} -{\Delta} e_{e,1}^{1} &= 0  \text{ in } {\Omega}_{1} , \\ e_{e,1}^{1} &= 0  \text{ on } {\partial{\Omega}_{1}^{0}} , \\ e_{e,1}^{1} &= u_{e}-{\lambda^{0}_{e}}  \text{ on } {\Gamma}_{12}\cup{\Gamma}_{41} . \end{aligned}\right. $$

Since ${\lambda ^{0}_{e}}$ is compatible with the even part of the Dirichlet boundary condition and $u_{e}\mid _{\partial {\Omega }_{1,\sigma }}\in H^{\frac {3}{2}}(\partial {\Omega }_{1,\sigma })$ for all $\sigma \in \mathcal {S}$, by Theorem 2, $e_{e,1}^{1}\in H^{2}({\Omega }_{1})$ exists and is unique. Moreover, we know that it is also continuous in $\overline {\Omega }_{1}$ due to Proposition 5.

In the same way, we solve in Ω_3_

$$ \left\{ \begin{aligned} -{\Delta} e_{e,3}^{1} &= 0  \text{ in } {\Omega}_{3} , \\ e_{e,3}^{1} &= 0  \text{ on } {\partial{\Omega}_{3}^{0}} , \\ e_{e,3}^{1} &= u_{e}-{\lambda^{0}_{e}}  \text{ on } {\Gamma}_{23}\cup{\Gamma}_{34} . \end{aligned}\right. $$

Since $u_{e}-{\lambda ^{0}_{e}}$ is even symmetric, it follows that the only solution $e_{e,3}^{1}\in H^{2}({\Omega }_{3})$ to this problem verifies $e_{e,3}^{1}(x,y) = e_{e,1}^{1}(-x,-y)$, for all $(x,y)\in \overline {\Omega }_{3}$.

**Iteration** ***k = 1***, Neumann step: Now, in Ω_2_, we solve

$$ \left\{ \begin{aligned} -{\Delta} e_{e,2}^{1} &= 0  \text{ in } {\Omega}_{2} , \\ e_{e,2}^{1} &= 0  \text{ on } {\partial{\Omega}_{2}^{0}} , \\ \partial_{x} e_{e,2}^{1} &= \partial_{x} e_{e,1}^{1} = -\partial_{x} (e_{e,1}^{1}(-x,y))\mid_{x=0}  \text{ on } {\Gamma}_{12} ,\\ \partial_{y} e_{e,2}^{1} &= \partial_{y} e_{e,3}^{1} = \partial_{y} (e_{e,1}^{1}(-x,-y))\mid_{y=0} = -\partial_{y} (e_{e,1}^{1}(-x,y))\mid_{y=0}  \text{ on } {\Gamma}_{23} . \end{aligned}\right. $$

This problem is well-posed in $H^{1}({\Omega }_{2})$. In addition, it is clear that the function defined in Ω_2_ by $(x,y)\mapsto -e_{e,1}^{1}(-x,y)$ solves the problem. By uniqueness, one deduces that the solution $e_{e,2}^{1}$ is given by $e_{e,2}^{1}(x,y) = -e_{e,1}^{1}(-x,y)$, for all (*x*,*y*) ∈Ω_2_. Note that this equality extends to the whole $\overline {\Omega }_{2}$. Again, in the exact same way, we get that the solution $e_{e,4}^{1}$ in Ω_4_ is given by $e_{e,4}^{1}(x,y) = -e_{e,1}^{1}(x,-y)$, for all $(x,y)\in \overline {\Omega }_{4}$.

We are left with a recombined error ${e_{e}^{1}}$ (defined in Ω ∖Γ) that is discontinuous across all parts of the skeleton Γ where $e_{e,1}^{1}\neq 0$ (see Fig. 3c). This may lead to discontinuities for the recombined solution ${u_{e}^{1}}=u_{e}+{e_{e}^{1}}$. Also note that ${e_{e}^{1}}$ is even symmetric in Ω ∖Γ.

**Iteration** ***k = 2***, Dirichlet step: In Ω_1_, we solve

$$ \left\{ \begin{aligned} -{\Delta} e_{e,1}^{2} &= 0  \text{ in } {\Omega}_{1} , \\ e_{e,1}^{2} &= 0  \text{ on } {\partial{\Omega}_{1}^{0}} , \\ e_{e,1}^{2} &= \theta e_{e,2}^{1}+(1-\theta)e_{e,1}^{1} = (1-2\theta)e_{e,1}^{1}  \text{ on } {\Gamma}_{12} ,\\ e_{e,1}^{2} &= (1-2\theta)e_{e,1}^{1}  \text{ on } {\Gamma}_{41} . \end{aligned}\right. $$

The unique solution to this problem is $e_{e,1}^{2} = (1-2\theta )e_{e,1}^{1}$. In the exact same way, we get in Ω_3_, $e_{e,3}^{2} = (1-2\theta )e_{e,3}^{1}$.

**Iteration** ***k = 2***, Neumann step: Now, in Ω_2_, we solve

$$ \left\{ \begin{aligned} -{\Delta} e_{e,2}^{2} &= 0  \text{ in } {\Omega}_{2} , \\ e_{e,2}^{2} &= 0  \text{ on } {\partial{\Omega}_{2}^{0}} , \\ \partial_{x} e_{e,2}^{2} &= \partial_{x} e_{e,1}^{2} = (1-2\theta)\partial_{x} e_{e,1}^{1}  \text{ on } {\Gamma}_{12} ,\\ \partial_{y} e_{e,2}^{2} &= \partial_{y} e_{e,3}^{2} = (1-2\theta)\partial_{y} e_{e,3}^{1}  \text{ on } {\Gamma}_{23} . \end{aligned} \right. $$

The unique solution to this problem is $e_{e,2}^{2} = (1-2\theta )e_{e,2}^{1}$. Again, in the exact same way, we get in Ω_4_, $e_{e,4}^{2} = (1-2\theta )e_{e,4}^{1}$. For all *𝜃* ∈ (0,1), the recombined solution ${e_{e}^{2}}$ is exactly $(1-2\theta ){e_{e}^{1}}$.

**Iterations** ***k ≥ 3***: From the analysis of the first two iterations, it follows by induction that, at iteration *k*, one has

$$ {e_{e}^{k}} = (1-2\theta)^{k-1} {e_{e}^{1}} \text{ in } {\Omega}\setminus{\Gamma} , $$

(see Fig. 3d). Therefore, the proposed domain decomposition method converges geometrically to the solution *u*_*e*_ both in the *L*^2^-norm and the broken *H*^1^-norm for all *𝜃* ∈ (0,1),

$$ \parallel u_{e} - {u_{e}^{k}}\parallel_{L^{2}({\Omega})}  \leq  C \mid1-2\theta\mid^{k-1} \text{and} {\sum}_{i\in \mathcal{I}} \parallel u_{e,i} - u_{e,i}^{k}\parallel_{H^{1}({\Omega}_{i})}  \leq  C^{\prime} \mid1-2\theta\mid^{k-1} . $$

Note that the recombined solution ${u_{e}^{k}}$ is in general not continuous across the skeleton Γ. □

#### *Remark 1*

It is actually not difficult to find an initial guess *λ*^0^ satisfying the assumptions of Theorem 6: the simplest way is to build a piecewise linear function on Γ. If we denote *P*_*k*_, *k* ∈{1,⋯ ,4}, the four boundary cross points, and set *λ*^0^(*P*_*k*_) := *g*(*P*_*k*_) for each *k*, then *λ*^0^ is compatible with the Dirichlet boundary condition. Next, we set $\lambda ^{0}(0,0):=\frac {1}{4}{\sum }_{k} \lambda ^{0}(P_{k})$ and perform a linear reconstruction on each interface Γ_*ij*_, which ensures that *λ*^0^ ∈ *C*^0^(Γ).

Of course, any other choice for *λ*^0^(0,0) would work as well, but this specific choice leads to a function *λ*^0^ with minimal slopes as it satisfies

$$ \frac{1}{4}{\sum}_{k} \lambda^{0}(P_{k}) = \arg \min_{\alpha}  {\sum}_{k} \mid \lambda^{0}(P_{k}) - \alpha \mid^{2} . $$

### Case of the odd symmetric part

Let us now turn to the odd symmetric part of problem (1). Given our initial guess, we obtain the following result.

#### **Theorem 7**

The Dirichlet-Neumann method applied to the odd symmetric part of problem (1) is not well-posed. More specifically, taking ${\lambda ^{0}_{o}}$ as the initial guess, there exists an integer *k*_0_ > 0 such that the solution to the problem obtained at the *k*_0_-th iteration is not unique. In addition, all possible solutions $u_{o}^{k_{0}}$ are singular at the cross-point, with a leading singularity of type $(\ln r)^{2}$.

The previous result shows that in this case, at some point, the Dirichlet-Neumann method is not applicable anymore. In practice, since we are able to find a solution to this ill-posed problem, one may wonder if it is possible to recover a nice behavior of the method if we go past this iteration *k*_0_. The next theorem provides a negative answer.

#### **Theorem 8**

If we let the Dirichlet-Neumann method go beyond the ill-posed iteration *k*_0_ from Theorem 7, we end up with a sequence $\{{u_{o}^{k}}\}_{k\geq k_{0}}$ of non-unique iterates. Moreover, for each *k* ≥ *k*_0_, all possible ${u_{o}^{k}}$ are singular at the cross-point, with a leading singularity of type $(\ln r)^{2(k-k_{0})+2}$.

**Fig. 2:**
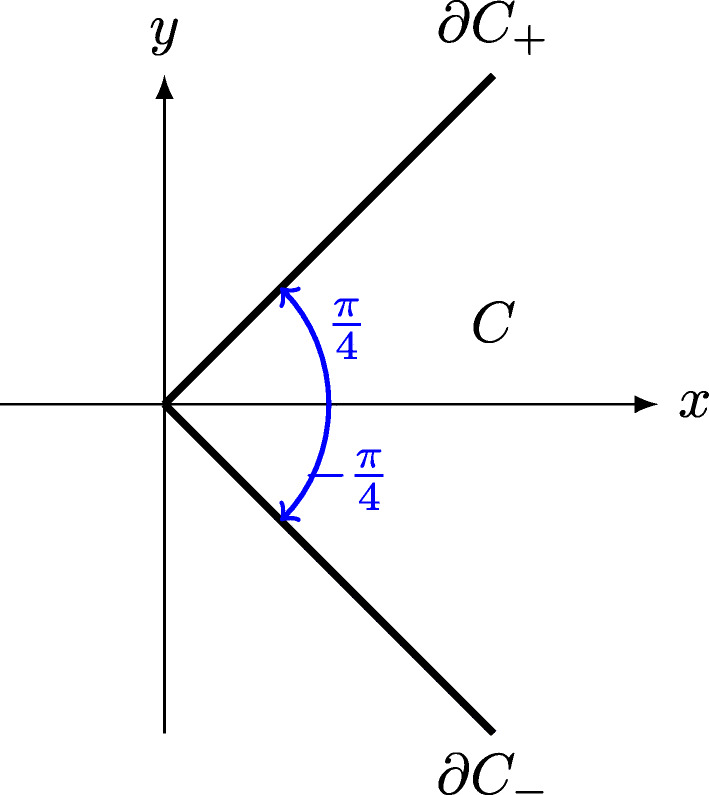
Cone *C* and its boundaries

Before giving the proofs of these theorems, we need four technical lemmas which provide solutions to the Laplace problem on the two-dimensional cone $C:=\mathbb {R}_{+}^{*}\times (-\frac {\pi }{4},\frac {\pi }{4})$ with different types of Dirichlet and Neumann boundary conditions on $\partial C_{-}:=\mathbb {R}_{+}^{*}\times \{-\frac {\pi }{4}\}$ and $\partial C_{+}:=\mathbb {R}_{+}^{*}\times \{\frac {\pi }{4}\}$ (see Fig. 2).

#### **Lemma 9**

For any integer *q* ≥ 0, there exist coefficients $(\alpha _{D,j,m}^{q})_{j,m}$ such that the function ${v_{D}^{q}}$ defined in polar coordinates for all (*r*,*ϕ*) ∈ *C* by

$$ {v_{D}^{q}}(r,\phi):= (\ln r)^{q} + {\sum}_{j,m=1}^{\lfloor \frac{q}{2} \rfloor} \alpha_{D,j,m}^{q} \phi^{2j}(\ln r)^{q-2m} $$

is in $L^{2}(C)\cap C^{\infty }\left (\overline { C}\setminus \{(0,0)\}\right )$ and solves

8 $$ \left\{ \begin{aligned} -{\Delta} v &= 0  \text{ in } C , \\ v &= (\ln r)^{q}  \text{ on } \partial C_{-}\cup \partial C_{+} . \end{aligned} \right.  $$

#### *Proof 4*

Let us proceed by induction.

**Base case.** The cases *q* = 0 and *q* = 1 are easily verified. Indeed, ${v_{D}^{0}}:=1$ and ${v_{D}^{1}}:=\ln r$ clearly solve (8) for *q* = 0 and *q* = 1, respectively.

**Induction step.** Let *q* ≥ 1 be fixed. We assume that the result of the lemma holds for all *ℓ* ≤ *q*. In order to find a solution to (8) for *q* + 1, we begin with the initial guess $(\ln r)^{q+1}$, which obviously satisfies the boundary condition. First, we compute a correction $r_{D}^{q+1}$ such that $(\ln r)^{q+1}+r_{D}^{q+1}$ is harmonic in *C*. Then, since the boundary conditions are no longer satisfied due to this first correction, we compute a second correction $\tilde {r}_{D}^{q+1}$ such that $(\ln r)^{q+1}+r_{D}^{q+1}+\tilde {r}_{D}^{q+1}$ solves (8).

For the first step, we start by computing the Laplacian in polar coordinates. Let us recall its expression for some smooth function *f* depending on (*r*,*ϕ*)

$$ {\Delta} f = \frac{\partial^{2} f}{\partial r^{2}} + \frac{1}{r}\frac{\partial f}{\partial r} + \frac{1}{r^{2}}\frac{\partial^{2} f}{\partial\phi^{2}} . $$

For our intial guess $(\ln r)^{q+1}$, this leads to

$$ {\Delta}\left( (\ln r)^{q+1} \right) = (q+1)q \frac{(\ln r)^{q-1}}{r^{2}}. $$

We can now cancel the term remaining on the right by adding on the left

$$ {\Delta}\left( (\ln r)^{q+1} - \frac{1}{2}\phi^{2}(q+1)q (\ln r)^{q-1} \right) = -\frac{1}{2}\phi^{2}(q+1)\cdots(q-2)\frac{(\ln r)^{q-3}}{r^{2}}. $$

Again, to remove the new term on the right, we add on the left

$$ \begin{aligned} {\Delta}\left( (\ln r)^{q+1} - \frac{1}{2}\phi^{2}(q+1)q (\ln r)^{q-1} + \right. & \left. \frac{1}{4!}\phi^{4}(q+1)\cdots(q-2)(\ln r)^{q-3}\right) \\ & = \frac{1}{4!}\phi^{4}(q+1)\cdots(q-4)\frac{(\ln r)^{q-5}}{r^{2}}, \end{aligned} $$

and continuing until the exponent on the $(\ln r)$ term in the right hand side reaches 0 or 1 gives

$$ {\Delta}\left( (\ln r)^{q+1} + {\sum}_{j=1}^{\lfloor\frac{q+1}{2}\rfloor} (-1)^{j}\frac{1}{(2j)!}\phi^{2j}(q+1)\cdots(q+2-2j)(\ln r)^{q+1-2j} \right) = 0 . $$

Therefore, introducing $\beta _{j}^{q+1}:=(-1)^{j}\binom {q+1}{2j}$ for all *j*, and defining the correction

$$ r_{D}^{q+1} := {\sum}_{j=1}^{\lfloor\frac{q+1}{2}\rfloor} \beta_{j}^{q+1}\phi^{2j}(\ln r)^{q+1-2j} , $$

we get a function $(\ln r)^{q+1}+r_{D}^{q+1}$ that is harmonic and is in $L^{2}(C)\cap C^{\infty }\left (\overline { C}\setminus \{(0,0)\}\right )$.

For the second step, let us note that this function verifies

$$ (\ln r)^{q+1}+r_{D}^{q+1} = (\ln r)^{q+1} + {\sum}_{j=1}^{\lfloor\frac{q+1}{2}\rfloor} \beta_{j}^{q+1}\left( \frac{\pi}{4}\right)^{2j}(\ln r)^{q+1-2j} $$

on *∂C*_−_∪ *∂C*_+_. From the induction hypothesis, we know that for each *j*, there exists a function $v_{D}^{q+1-2j}$ that solves (8) for *q* + 1 − 2*j*. Therefore, if we define

$$ \tilde{r}_{D}^{q+1} := -{\sum}_{j=1}^{\lfloor\frac{q+1}{2}\rfloor} \beta_{j}^{q+1}\left( \frac{\pi}{4}\right)^{2j}v_{D}^{q+1-2j} , $$

we obtain a function $v_{D}^{q+1}:=(\ln r)^{q+1}+r_{D}^{q+1}+\tilde {r}_{D}^{q+1}$ that solves (8) for *q* + 1 and is in $L^{2}(C)\cap C^{\infty }\left (\overline { C}\setminus \{(0,0)\}\right )$. In addition, given the expressions of the corrections $r_{D}^{q+1}$ and $\tilde {r}_{D}^{q+1}$, it follows that $v_{D}^{q+1}$ can be written as

$$ v_{D}^{q+1}(r,\phi) = (\ln r)^{q+1} + {\sum}_{j,m=1}^{\lfloor \frac{q+1}{2} \rfloor} \alpha_{D,j,m}^{q+1} \phi^{2j}(\ln r)^{q+1-2m} , $$

where the coefficients $\alpha _{D,j,m}^{q+1}$ depend on the $\beta _{j}^{q+1}$ and the $\alpha _{D,j,m}^{\ell }$, for *ℓ* ≤ *q* − 1. □

#### **Lemma 10**

For any integer *q* ≥ 0, there exist coefficients $(\alpha _{D^{\prime },j,m}^{q})_{j,m}$ such that the function $v_{D^{\prime }}^{q}$ defined in polar coordinates for all (*r*,*ϕ*) ∈ *C* by

$$ v_{D^{\prime}}^{q}(r,\phi):= \frac{4}{\pi}\phi(\ln r)^{q} + {\sum}_{j,m=1}^{\lfloor \frac{q}{2} \rfloor} \alpha_{D^{\prime},j,m}^{q} \phi^{2j+1}(\ln r)^{q-2m} $$

is in $L^{2}(C)\cap C^{\infty }\left (\overline { C}\setminus \{(0,0)\}\right )$ and solves

9 $$ \left\{ \begin{aligned} -{\Delta} v &= 0  \text{ in } C , \\ v &= -(\ln r)^{q}  \text{ on } \partial C_{-} , \\ v &= (\ln r)^{q}  \text{ on } \partial C_{+} . \end{aligned} \right.  $$

#### *Proof 5*

Let us proceed by induction, in the same way as in the proof of Lemma 9.

**Base case.** It is not difficult to check that $v_{D^{\prime }}^{0}:=\frac {4}{\pi }\phi $ and $v_{D^{\prime }}^{1}:=\frac {4}{\pi }\phi \ln r$ solve (9) for *q* = 0 and *q* = 1, respectively.

**Induction step.** Let *q* ≥ 1 be fixed. We assume that the result of the lemma holds for all *ℓ* ≤ *q*. As for Lemma 9, in order to find a solution to (9) for *q* + 1, we proceed in two steps, this time starting from $\frac {4}{\pi }\phi (\ln r)^{q+1}$ as initial guess.

For the first step, following a similar iterative approach, we obtain a correction

$$ r_{D^{\prime}}^{q+1} := \frac{4}{\pi}\phi r_{D}^{q+1} = {\sum}_{j=1}^{\lfloor\frac{q+1}{2}\rfloor} \beta_{j}^{q+1}\phi^{2j+1}(\ln r)^{q+1-2j} , $$

where $\beta _{j}^{q+1}:=\frac {4}{\pi }(-1)^{j}\binom {q+1}{2j}$ for all *j*. This enables us to get a function $\frac {4}{\pi }\phi (\ln r)^{q+1}+r_{D^{\prime }}^{q+1}$ that is harmonic and is in $L^{2}(C)\cap C^{\infty }\left (\overline { C}\setminus \{(0,0)\}\right )$. However, we have

$$ \frac{4}{\pi}\phi(\ln r)^{q+1}+r_{D^{\prime}}^{q+1} = \pm \left( (\ln r)^{q+1} + {\sum}_{j=1}^{\lfloor\frac{q+1}{2}\rfloor} \beta_{j}^{q+1}\left( \frac{\pi}{4}\right)^{2j+1}(\ln r)^{q+1-2j} \right) $$

on *∂C*_±_. This notation means that the sign depends on which part of the boundary is considered: + on *∂C*_+_ and − on *∂C*_−_. Therefore, using the induction hypothesis, we introduce a second correction

$$ \tilde{r}_{D^{\prime}}^{q+1} := -{\sum}_{j=1}^{\lfloor\frac{q+1}{2}\rfloor} \beta_{j}^{q+1}\left( \frac{\pi}{4}\right)^{2j+1}v_{D^{\prime}}^{q+1-2j} , $$

such that $v_{D^{\prime }}^{q+1}:=\frac {4}{\pi }\phi (\ln r)^{q+1}+r_{D^{\prime }}^{q+1}+\tilde {r}_{D^{\prime }}^{q+1}$ solves (9) for *q* + 1 and is in $L^{2}(C)\cap C^{\infty }\left (\overline { C}\setminus \{(0,0)\}\right )$. In addition, this function can be written as

$$ v_{D^{\prime}}^{q+1}(r,\phi) = \frac{4}{\pi}\phi(\ln r)^{q+1} + {\sum}_{j,m=1}^{\lfloor \frac{q+1}{2} \rfloor} \alpha_{D^{\prime},j,m}^{q+1} \phi^{2j+1}(\ln r)^{q+1-2m} , $$

where the coefficients $\alpha _{D^{\prime },j,m}^{q+1}$ depend on the $\beta _{j}^{q+1}$ and the $\alpha _{D^{\prime },j,m}^{\ell }$, for *ℓ* ≤ *q* − 1. □

#### **Lemma 11**

For any integer *q* ≥ 0, there exist coefficients $(\alpha _{N,j,m}^{q})_{j,m}$ such that the function ${v_{N}^{q}}$ defined in polar coordinates for all (*r*,*ϕ*) ∈ *C* by

$$ {v_{N}^{q}}(r,\phi):= \phi(\ln r)^{q} + {\sum}_{j,m=1}^{\lfloor \frac{q}{2} \rfloor} \alpha_{N,j,m}^{q} \phi^{2j+1}(\ln r)^{q-2m} $$

is in $L^{2}(C)\cap C^{\infty }\left (\overline { C}\setminus \{(0,0)\}\right )$ and solves

10 $$ \left\{ \begin{aligned} -{\Delta} v &= 0  \text{ in } C , \\ \partial_{\phi} v &= (\ln r)^{q}  \text{ on } \partial C_{-}\cup \partial C_{+} . \end{aligned} \right.  $$

#### *Proof 6*

Again, we proceed by induction, in the same spirit as in the proofs of the previous lemmas.

**Base case** Obviously, ${v_{N}^{0}}:=\phi $ and ${v_{N}^{1}}:=\phi \ln r$ solve (10) for *q* = 0 and *q* = 1, respectively.

**Induction step** Let *q* ≥ 1 be fixed. The result of the lemma is assumed to hold for all *ℓ* ≤ *q*. As before, we aim at finding a solution to (10) for *q* + 1 by proceeding in two steps, this time starting from $\phi (\ln r)^{q+1}$ as initial guess.

This initial guess is the same as in the proof of Lemma 10 (up to a factor $\frac {4}{\pi }$); therefore, we have for the first correction

$$ r_{N}^{q+1} := \phi r_{D}^{q+1} = {\sum}_{j=1}^{\lfloor\frac{q+1}{2}\rfloor} \beta_{j}^{q+1}\phi^{2j+1}(\ln r)^{q+1-2j} , $$

where $\beta _{j}^{q+1}:=(-1)^{j}\binom {q+1}{2j}$ for all *j*. This choice ensures that $\phi (\ln r)^{q+1}+r_{N}^{q+1}$ is harmonic and is in $L^{2}(C)\cap C^{\infty }\left (\overline { C}\setminus \{(0,0)\}\right )$. However, the boundary condition satisfied by this new function is

$$ \partial_{\phi} \left( \phi(\ln r)^{q+1}+r_{N}^{q+1} \right) =    (\ln r)^{q+1} + {\sum}_{j=1}^{\lfloor\frac{q+1}{2}\rfloor} (2j+1) \beta_{j}^{q+1}\left( \frac{\pi}{4}\right)^{2j}(\ln r)^{q+1-2j} $$

on *∂C*_−_∪ *∂C*_+_. This time, the induction hypothesis leads to a second correction

$$ \tilde{r}_{N}^{q+1} := -{\sum}_{j=1}^{\lfloor\frac{q+1}{2}\rfloor} (2j+1) \beta_{j}^{q+1}\left( \frac{\pi}{4}\right)^{2j}v_{N}^{q+1-2j} . $$

Finally, we define $v_{N}^{q+1}:=\phi (\ln r)^{q+1}+r_{N}^{q+1}+\tilde {r}_{N}^{q+1}$, which is solution to (10) for *q* + 1 and is in $L^{2}(C)\cap C^{\infty }\left (\overline { C}\setminus \{(0,0)\}\right )$. Moreover, we have for this function the expression

$$ v_{N}^{q+1}(r,\phi) = \phi(\ln r)^{q+1} + {\sum}_{j,m=1}^{\lfloor \frac{q+1}{2} \rfloor} \alpha_{N,j,m}^{q+1} \phi^{2j+1}(\ln r)^{q+1-2m} , $$

where the coefficients $\alpha _{N,j,m}^{q+1}$ depend on the $\beta _{j}^{q+1}$ and the $\alpha _{N,j,m}^{\ell }$, for *ℓ* ≤ *q* − 1. □

#### **Lemma 12**

For any integer *q* ≥ 0, there exist coefficients $(\alpha _{N^{\prime },j,m}^{q})_{j,m}$ such that the function $v_{N^{\prime }}^{q}$ defined in polar coordinates for all (*r*,*ϕ*) ∈ *C* by

$$ \begin{aligned} v_{N^{\prime}}^{q}(r,\phi)&:= \frac{4}{\pi}\left( \frac{1}{2}\phi^{2}(\ln r)^{q}-\frac{1}{(q+1)(q+2)}(\ln r)^{q+2}\right)\\ &\quad + {\sum}_{j,m=2}^{\lfloor \frac{q}{2}\rfloor+1} \alpha_{N^{\prime},j,m}^{q} \phi^{2j}(\ln r)^{q+2-2m} \end{aligned} $$

is in $L^{2}(C)\cap C^{\infty }\left (\overline { C}\setminus \{(0,0)\}\right )$ and solves

11 $$ \left\{ \begin{aligned} -{\Delta} v &= 0  \text{ in } C , \\ \partial_{\phi} v &= -(\ln r)^{q}  \text{ on } \partial C_{-} , \\ \partial_{\phi} v &= (\ln r)^{q}  \text{ on } \partial C_{+} . \end{aligned} \right.  $$

#### *Proof 7*

We keep proceeding by induction.

**Base case** Take *q* = 0. Then we have $v_{N^{\prime }}^{0}:=\frac {2}{\pi }\left (\phi ^{2}-(\ln r)^{2} \right )$, which is indeed solution to (11) for *q* = 0.

**Induction step** Let *q* ≥ 1 be fixed. The result of the lemma is assumed to hold for all *ℓ* ≤ *q*. In order to find a solution to (11) for *q* + 1, we follow the usual two steps starting from the initial guess $\frac {2}{\pi }\phi ^{2}(\ln r)^{q+1}$, which satisfies the boundary conditions.

For the first step, we reuse the computations performed in the proof of Lemma 9, replacing *q* + 1 by *q* + 3. Rewriting the function $(\ln r)^{q+3}+r_{D}^{q+3}$, we get that

$$ (\ln r)^{q+3}-\frac{1}{2}\phi^{2}(q+3)(q+2)(\ln r)^{q+1}+{\sum}_{j=2}^{\lfloor\frac{q+3}{2}\rfloor} (-1)^{j}\binom{q+3}{2j}\phi^{2j}(\ln r)^{q+3-2j} $$

is a harmonic function. We can easily deduce from this a first correction for our initial guess,

$$ r_{N^{\prime}}^{q+1} := -\frac{4}{\pi}\frac{1}{(q+3)(q+2)}(\ln r)^{q+3} + {\sum}_{j=2}^{\lfloor\frac{q+3}{2}\rfloor} \beta_{j}^{q+1}\phi^{2j}(\ln r)^{q+3-2j} , $$

where $\beta _{j}^{q+1}:=\frac {4}{\pi }\frac {(-1)^{j+1}}{(q+3)(q+2)}\binom {q+3}{2j}$ for all *j*. As desired, the new function $\frac {2}{\pi }\phi ^{2}(\ln r)^{q+1}+r_{N^{\prime }}^{q+1}$ is harmonic and belongs to $L^{2}(C)\cap C^{\infty }\left (\overline { C}\setminus \{(0,0)\}\right )$. In addition, it satisfies

$$ \partial_{\phi} \left( \frac{2}{\pi}\phi^{2}(\ln r)^{q+1}+r_{N^{\prime}}^{q+1} \right) = \pm \left( (\ln r)^{q+1} + {\sum}_{j=2}^{\lfloor\frac{q+3}{2}\rfloor} (2j) \beta_{j}^{q+1}\left( \frac{\pi}{4}\right)^{2j-1}(\ln r)^{q+3-2j} \right) $$

on *∂C*_±_. Again, using the induction hypothesis for all *ℓ* ≤ *q* − 1, we obtain a second correction

$$ \tilde{r}_{N^{\prime}}^{q+1} := -{\sum}_{j=2}^{\lfloor\frac{q+3}{2}\rfloor} (2j) \beta_{j}^{q+1}\left( \frac{\pi}{4}\right)^{2j-1}v_{N^{\prime}}^{q+3-2j} . $$

Therefore, we are able to build a function $v_{N^{\prime }}^{q+1}:=\frac {2}{\pi }\phi ^{2}(\ln r)^{q+1}+r_{N^{\prime }}^{q+1}+\tilde {r}_{N^{\prime }}^{q+1}$ that solves (11) for *q* + 1 and is in $L^{2}(C)\cap C^{\infty }\left (\overline { C}\setminus \{(0,0)\}\right )$, namely

$$ \begin{aligned} v_{N^{\prime}}^{q+1}(r,\phi) = & \frac{4}{\pi}\left( \frac{1}{2}\phi^{2}(\ln r)^{q+1}-\frac{1}{(q+2)(q+3)}(\ln r)^{q+3}\right) \\ & + {\sum}_{j,m=2}^{\lfloor \frac{q+1}{2}\rfloor+1} \alpha_{N^{\prime},j,m}^{q+1} \phi^{2j}(\ln r)^{q+3-2m} , \end{aligned} $$

where the coefficients $\alpha _{N^{\prime },j,m}^{q+1}$ depend on the $\beta _{j}^{q+1}$ and the $\alpha _{N^{\prime },j,m}^{\ell }$, for *ℓ* ≤ *q* − 1. □

We can now prove Theorem 7 and Theorem 8.

#### *Proof 8* (Proof of Theorem 7)

We want to prove that the algorithm has a nice behavior up to some iteration *k*_0_, where the iterate becomes non-unique and singular near the cross-point, with a leading singularity of type $(\ln r)^{2}$. The idea is to show that, in general, after two iterations only, the approximate solution given by the method is singular near the cross-point. In order to do so, let us apply the Dirichlet-Neumann method step by step to (7), and write the local subproblems in terms of the local errors $e_{o,i}^{k}$.

**Iteration** ***k = 1***, Dirichlet step: In Ω_1_, we solve

$$ \left\{\begin{aligned} -{\Delta} e_{o,1}^{1} &= 0  \text{ in } {\Omega}_{1} , \\ e_{o,1}^{1} &= 0  \text{ on } {\partial{\Omega}_{1}^{0}} , \\ e_{o,1}^{1} &= u_{o}-{\lambda^{0}_{o}}  \text{ on } {\Gamma}_{12}\cup{\Gamma}_{41} . \end{aligned} \right. $$

As for the even symmetric case, by Theorem 2, $e_{o,1}^{1}\in H^{2}({\Omega }_{1})\subset C^{0}(\overline {\Omega }_{1})$ exists and is unique. In the same way, we solve in Ω_3_

$$ \left\{ \begin{aligned} -{\Delta} e_{o,3}^{1} &= 0  \text{ in } {\Omega}_{3} , \\ e_{o,3}^{1} &= 0  \text{ on } {\partial{\Omega}_{3}^{0}} , \\ e_{o,3}^{1} &= u_{o}-{\lambda^{0}_{o}}  \text{ on } {\Gamma}_{23}\cup{\Gamma}_{34} . \end{aligned}\right. $$

Since $u_{o}-{\lambda ^{0}_{o}}$ is odd symmetric, it follows that the unique solution $e_{o,3}^{1}$ to this problem verifies, for all $(x,y)\in \overline {\Omega }_{3}$, $e_{o,3}^{1}(x,y) = -e_{o,1}^{1}(-x,-y)$. Note that the recombined solution is continuous across the cross-point from Ω_1_ to Ω_3_ because $e_{o,1}^{1}(0,0)=(u_{o}-{\lambda ^{0}_{o}})(0,0)=0$, since $(u_{o}-{\lambda ^{0}_{o}})$ is odd symmetric (see Fig. 5b).

**Iteration** ***k = 1***, Neumann step: Now, in Ω_2_, we solve

$$ \left\{ \begin{aligned} -{\Delta} e_{o,2}^{1} &= 0  \text{ in } {\Omega}_{2} , \\ e_{o,2}^{1} &= 0  \text{ on } {\partial{\Omega}_{2}^{0}} , \\ \partial_{x} e_{o,2}^{1} &= \partial_{x} e_{o,1}^{1} = -\partial_{x} (e_{o,1}^{1}(-x,y))\mid_{x=0}  \text{ on } {\Gamma}_{12} ,\\ \partial_{y} e_{o,2}^{1} &= \partial_{y} e_{o,3}^{1} = -\partial_{y} (e_{o,1}^{1}(-x,-y))\mid_{y=0} = \partial_{y} (e_{o,1}^{1}(-x,y))\mid_{y=0}  \text{ on } {\Gamma}_{23} . \end{aligned}\right. $$

Here again, we know the problem is well-posed in $H^{1}({\Omega }_{2})$. However, in contrast to the even symmetric case, we cannot argue that $\pm e_{o,1}^{1}(-x,y)$ solves this problem. There is a sign incompatibility in the boundary condition: minus sign on Γ_12_ and plus sign on Γ_23_. The solution $e_{o,2}^{1}$ cannot be expressed explicitly. Nevertheless, since $e_{o,1}^{1}\in H^{2}({\Omega }_{1})$, we know that the trace $e_{o,1}^{1}(\cdot ,-1)$ on the bottom side of Ω_1_ and the normal derivative $\partial _{x} e_{o,1}^{1}(0,\cdot )$ on the right side of Ω_1_ satisfy the compatibility relation in Theorem 4 at the mixed corner (0,− 1). Therefore, as $e_{o,1}^{1}(\cdot ,-1)=0$, this compatibility relation is also satisfied by the boundary conditions enforced in the previous problem at this same corner (0,− 1) in Ω_2_. The same argument can be used for the other mixed corner (1,0). Moreover, the *H*^2^ regularity of $e_{o,1}^{1}$ also implies that $\partial _{x} e_{o,1}^{1}\in H^{\frac {1}{2}}({\Gamma }_{12})$ and $\partial _{y} e_{o,3}^{1}\in H^{\frac {1}{2}}({\Gamma }_{23})$, which finally yields $e_{o,2}^{1}\in H^{2}({\Omega }_{2})\subset C^{0}(\overline {\Omega }_{2})$ by Theorem 4 and Proposition 5. Despite this additional regularity property, we are still not able to express the solution. Especially, we do not know the value of $e_{o,2}^{1}$ at (0,0). For the reader’s convenience, let us denote it by $\delta ^{1}:=e_{o,2}^{1}(0,0)$. Then, in Ω_4_, we get that the solution $e_{o,4}^{1}$ is given by $e_{o,4}^{1}(x,y) = -e_{o,2}^{1}(-x,-y)$, for all $(x,y)\in \overline {\Omega }_{4}$. Therefore, the recombined solution jumps across the cross-point from *δ*^1^ in Ω_2_ to − *δ*^1^ in Ω_4_. This time, we are left with a recombined error ${e_{o}^{1}}$ that is odd symmetric in Ω ∖Γ, and discontinuous across the cross point (see Fig. 5b).

**Iteration** ***k = 2***, Dirichlet step: In Ω_1_, we solve

12 $$ \left\{ \begin{aligned} -{\Delta} e_{o,1}^{2} &= 0  \text{ in } {\Omega}_{1} , \\ e_{o,1}^{2} &= 0  \text{ on } {\partial{\Omega}_{1}^{0}} , \\ e_{o,1}^{2} &= \theta e_{o,2}^{1}+(1-\theta)e_{o,1}^{1}  \text{ on } {\Gamma}_{12} , \\ e_{o,1}^{2} &= \theta e_{o,4}^{1}+(1-\theta)e_{o,1}^{1}  \text{ on } {\Gamma}_{41} . \end{aligned}\right.  $$

Since the boundary conditions are continuous on $\overline {\Gamma }_{12}$ and $\overline {\Gamma }_{41}$, we are able to compute their limits at the cross-point, which yields

$$ \begin{aligned} \lim_{\underset{(x,y)\in{\Gamma}_{12}}{(x,y)\to(0,0)}} \left( \theta e_{o,2}^{1}+(1-\theta)e_{o,1}^{1}\right)(x,y) &= \theta\delta^{1} , \\ \lim_{\underset{(x,y)\in{\Gamma}_{41}}{(x,y)\to(0,0)}} \left( \theta e_{o,4}^{1}+(1-\theta)e_{o,1}^{1}\right)(x,y) &= -\theta\delta^{1} . \end{aligned} $$

In other words, whenever *δ*^1^≠ 0, the method enforces a discontinuous Dirichlet boundary condition at this step, which may lead to a singular solution since the compatibility relation (3) is no longer satisfied. Especially, the problem is not necessarily well-posed so the Dirichlet-Neumann method might not even be valid in this case. Note that there is no other discontinuity enforced since the boundary conditions on Γ_12_ and Γ_41_ extend to 0 at $\overline {\Gamma }_{12}\cap \overline {\Gamma }_{1}$ and $\overline {\Gamma }_{41}\cap \overline {\Gamma }_{1}$.

**Case** ***δ***^***1***^***≠ 0*** In what follows, we prove existence and uniqueness of $e_{o,1}^{2}$, exhibiting the type of singularity induced by this nonsmooth boundary condition. In order to do so, we try to decompose $e_{o,1}^{2}$ as the sum of a regular part ${v_{1}^{2}}$ and a singular part ${w_{1}^{2}}$, in the same spirit as in [37] for more regular problems. First, for each $i\in \mathcal {I}$, let us introduce the angle *ϕ*_*i*_ ∈ (0,2*π*) such that the rotation *R*_*i*_ of angle − *ϕ*_*i*_, given in polar coordinates by

$$ R_{i} : (r,\phi) \mapsto \left( r,\phi-\phi_{i}\right) , $$

maps the quadrant containing Ω_*i*_ onto the cone *C*. More specifically, we have

$$ \phi_{1} := \frac{5\pi}{4} ,  \phi_{2} := \frac{7\pi}{4} ,  \phi_{3} := \frac{\pi}{4} ,  \phi_{4} := \frac{3\pi}{4} . $$

Using these notations, we define ${w_{1}^{2}}:=(\theta \delta ^{1}) \cdot v^{0}_{D^{\prime }}\circ R_{1}$, whose expression in polar coordinates (*r*,*ϕ*) reads

13 $$ {w_{1}^{2}}(r,\phi) = \theta\delta^{1} \frac{4}{\pi}\left( \phi-\phi_{1}\right) .  $$

We know from Lemma 10 that ${w_{1}^{2}}$ is of class $C^{\infty }$ in $(\mathbb {R}_{+})^{2}\setminus \{(0,0)\}$, and that it satisfies

$$ \left\{ \begin{aligned} -{\Delta} {w_{1}^{2}} &= 0  \text{ in } \mathbb{R}^{*}_{-}\times \mathbb{R}^{*}_{-} , \\ {w_{1}^{2}} &= \theta \delta^{1}  \text{ on } \{0\}\times\mathbb{R}^{*}_{-} , \\ {w_{1}^{2}} &= -\theta \delta^{1}  \text{ on } \mathbb{R}^{*}_{-}\times\{0\} . \end{aligned} \right. $$

Then, since we would like ${v_{1}^{2}}+{w_{1}^{2}}$ to solve (12), we must define ${v_{1}^{2}}$ such that

$$ \left\{ \begin{aligned} -{\Delta} {v_{1}^{2}} &= 0  \text{ in } {\Omega}_{1} , \\ {v_{1}^{2}} &= -{w_{1}^{2}}  \text{ on } {\partial{\Omega}_{1}^{0}} , \\ {v_{1}^{2}} &= \theta e_{o,2}^{1}+(1-\theta)e_{o,1}^{1}-{w_{1}^{2}}  \text{ on } {\Gamma}_{12} , \\ {v_{1}^{2}} &= \theta e_{o,4}^{1}+(1-\theta)e_{o,1}^{1}-{w_{1}^{2}}  \text{ on } {\Gamma}_{41} . \end{aligned} \right. $$

Note that the Dirichlet boundary condition enforced here is in $C^{0}(\partial {\Omega }_{1})\cap H^{\frac {1}{2}}(\partial {\Omega }_{1,\sigma })$ for all $\sigma \in \mathcal {S}$. Therefore, ${v_{1}^{2}}\in H^{1}({\Omega }_{1})$ exists and is unique. Therefore, we have built (in a unique way) a solution ${v_{1}^{2}}+{w_{1}^{2}}$ to (12) which is in $L^{2}({\Omega }_{1})\setminus H^{1}({\Omega }_{1})$. Indeed, it is easy to show that ${w_{1}^{2}}$ is in $L^{2}({\Omega }_{1})$, but not in $H^{1}({\Omega }_{1})$ since

$$ \mid\nabla {w_{1}^{2}}\mid^{2} = \left( \theta\delta^{1}\frac{4}{\pi}\right)^{2}\frac{1}{r^{2}} . $$

Now, in order to conclude that $e_{o,1}^{2}={v_{1}^{2}}+{w_{1}^{2}}$, we must have uniqueness of the solution to (12) in $L^{2}({\Omega }_{1})$. This uniqueness property is indeed guaranteed since we know from [37, Theorem 4.4.3.3] that the subspace of all solutions $z\in L^{2}({\Omega }_{1})$ to

$$ \left\{ \begin{aligned} -{\Delta} z &= 0  \text{ in } {\Omega}_{1} , \\ z &= 0  \text{ on } \partial{\Omega}_{1} , \end{aligned}\right. $$

is of dimension 0. Hence, $e_{o,1}^{2}:={v_{1}^{2}}+{w_{1}^{2}}$ exists and is unique.

In the same way, one can conclude that, in Ω_3_, $e_{o,3}^{2}:={v_{3}^{2}}+{w_{3}^{2}}$ exists and is unique, with ${v_{3}^{2}}\in H^{1}({\Omega }_{3})$ and ${w_{3}^{2}}\in L^{2}({\Omega }_{3})\setminus H^{1}({\Omega }_{3})$. Of course, ${v_{3}^{2}}$ and ${w_{3}^{2}}$ can be obtained immediately from ${v_{1}^{2}}$ and ${w_{1}^{2}}$ using symmetry arguments, which gives for ${w_{3}^{2}}$

14 $$ {w_{3}^{2}}(r,\phi) = -\theta\delta^{1} \frac{4}{\pi}\left( \phi-\phi_{3} \right) .  $$

It is now clear that the algorithm generates a singular solution at this step. In order to estimate how the singularity propagates in the Neumann step, let us keep going and see what happens.

**Iteration** ***k = 2***, Neumann step: In Ω_2_, we solve

15 $$ \left\{ \begin{aligned} -{\Delta} e_{o,2}^{2} &= 0  \text{ in } {\Omega}_{2} , \\ e_{o,2}^{2} &= 0  \text{ on } {\partial{\Omega}_{2}^{0}} , \\ \partial_{x} e_{o,2}^{2} &= \partial_{x} e_{o,1}^{2}  \text{ on } {\Gamma}_{12} ,\\ \partial_{y} e_{o,2}^{2} &= \partial_{y} e_{o,3}^{2}  \text{ on } {\Gamma}_{23} . \end{aligned}\right.  $$

Due to the lack of regularity of $e_{o,1}^{2}$ and $e_{o,3}^{2}$, standard results fail to apply, so that existence and uniqueness of $e_{o,2}^{2}$ are not guaranteed. As in subdomains Ω_1_ and Ω_3_, we decompose $e_{o,2}^{2}$ as the sum of a regular part ${v_{2}^{2}}$ and a singular part ${w_{2}^{2}}$, exhibiting the singularity of ${w_{2}^{2}}$. Using the previous decompositions for $e_{o,1}^{2}$ and $e_{o,3}^{2}$, we rewrite the boundary conditions on Γ_12_ and Γ_23_,

$$ \left\{ \begin{aligned} \partial_{x} e_{o,2}^{2} &= \partial_{x} {v_{1}^{2}} + \partial_{x} {w_{1}^{2}}  \text{ on } {\Gamma}_{12} ,\\ \partial_{y} e_{o,2}^{2} &= \partial_{y} {v_{3}^{2}} + \partial_{y} {w_{3}^{2}}  \text{ on } {\Gamma}_{23} . \end{aligned}\right. $$

Then, let us introduce the function ${w_{2}^{2}}:=-(\theta \delta ^{1}\frac {4}{\pi }) \cdot v^{0}_{N^{\prime }}\circ R_{2}$, given in polar coordinates by

16 $$ {w_{2}^{2}}(r,\phi) = -\theta\delta^{1}\frac{8}{\pi^{2}}\left[ \left( \phi-\phi_{2} \right)^{2} - (\ln r)^{2} \right] .  $$

From Lemma 12, we know that ${w_{2}^{2}}\in C^{\infty }(\mathbb {R}_{+}\times \mathbb {R}_{-}\setminus \{(0,0)\})$, and that it satisfies

$$ \left\{ \begin{aligned} -{\Delta} {w_{2}^{2}} &= 0  \text{ in } \mathbb{R}^{*}_{+}\times \mathbb{R}^{*}_{-} , \\ \partial_{x} {w_{2}^{2}} &= \partial_{x} {w_{1}^{2}} = -\left( \theta\delta^{1}\frac{4}{\pi}\right)\frac{1}{y}  \text{ on } \{0\}\times\mathbb{R}^{*}_{-} , \\ \partial_{y} {w_{2}^{2}} &= \partial_{y} {w_{3}^{2}} = -\left( \theta\delta^{1}\frac{4}{\pi}\right)\frac{1}{x}  \text{ on } \mathbb{R}^{*}_{+}\times\{0\} . \end{aligned}\right. $$

This time, in order for ${v_{2}^{2}}+{w_{2}^{2}}$ to solve the problem for $e_{o,2}^{2}$, we define ${v_{2}^{2}}$ such that

$$ \left\{ \begin{aligned} -{\Delta} {v_{2}^{2}} &= 0  \text{ in } {\Omega}_{2} , \\ {v_{2}^{2}} &= -{w_{2}^{2}}  \text{ on } {\partial{\Omega}_{2}^{0}} , \\ \partial_{x} {v_{2}^{2}} &= \partial_{x} {v_{1}^{2}}  \text{ on } {\Gamma}_{12} ,\\ \partial_{y} {v_{2}^{2}} &= \partial_{y} {v_{3}^{2}}  \text{ on } {\Gamma}_{23} . \end{aligned}\right. $$

Given the regularities of ${w_{2}^{2}}$, ${v_{1}^{2}}$, and ${v_{3}^{2}}$, we deduce that ${v_{2}^{2}}$ exists and is unique in $H^{1}({\Omega }_{2})$. Again, we have built (in a unique way) a solution ${v_{2}^{2}}+{w_{2}^{2}}$ to (15), where ${v_{2}^{2}}\in H^{1}({\Omega }_{2})$ and ${w_{2}^{2}}\in L^{2}({\Omega }_{2})\setminus H^{1}({\Omega }_{2})$. But this is not enough to conclude that $e_{o,2}^{2}={v_{2}^{2}}+{w_{2}^{2}}$. As for Ω_1_, we know that this last equality holds provided that the subspace of all solutions $z\in L^{2}({\Omega }_{2})$ to

$$ \left\{ \begin{aligned} -{\Delta} z &= 0  \text{ in } {\Omega}_{2} , \\ z &= 0  \text{ on } {\partial{\Omega}_{2}^{0}} , \\ \partial_{n} z &= 0  \text{ on } {\Gamma}_{12}\cup{\Gamma}_{23} , \end{aligned}\right. $$

is of dimension 0. Unfortunately, it follows from [37, Theorem 4.4.3.3] that its dimension is 1, and that it is spanned by a function in $L^{2}({\Omega }_{2})$, say *z*_2_, that admits a singularity of type $\ln r$ at the cross-point. Therefore, one has that $e_{o,2}^{2}$ is not unique, and it can be written as $e_{o,2}^{2}={v_{2}^{2}}+{w_{2}^{2}}+{C_{2}^{2}}z_{2}$, for some constant ${C_{2}^{2}}\in \mathbb {R}$. However, no matter the value of ${C_{2}^{2}}$, one may always deduce that, in a neighborhood $\mathcal {V}_{0}$ of (0,0),

17 $$ e_{o,2}^{2} \simeq \theta\delta^{1}\frac{8}{\pi^{2}}(\ln r)^{2}  \text{ in } {\Omega}_{2}\cap\mathcal{V}_{0} .  $$

Note that there are actually three singular terms in $e_{o,2}^{2}$: (*ϕ* − *ϕ*_2_)^2^, $(\ln r)^{2}$ and $\ln r$. So the leading singularity is indeed $(\ln r)^{2}$.

Besides, one gets a similar result for $e_{o,4}^{2}$. That is $e_{o,4}^{2}$ is not unique and can be written as $e_{o,4}^{2}={v_{4}^{2}}+{w_{4}^{2}}+{C_{4}^{2}}z_{4}$ for some ${C_{4}^{2}}\in \mathbb {R}$, where ${v_{4}^{2}}\in H^{1}({\Omega }_{4})$, ${w_{4}^{2}}\in L^{2}({\Omega }_{4})\setminus H^{1}({\Omega }_{4})$ is given by

18 $$ {w_{4}^{2}}(r,\phi) = \theta\delta^{1}\frac{8}{\pi^{2}}\left[ \left( \phi-\phi_{4} \right)^{2} - (\ln r)^{2} \right] ,  $$

and $z_{4}\in L^{2}({\Omega }_{4})$ admits a singularity of type $\ln r$ near the cross-point, Of course, one obtains a similar asymptotic result in the neighborhood $\mathcal {V}_{0}$ of (0,0), i.e.,

$$ e_{o,4}^{2} \simeq -\theta\delta^{1}\frac{8}{\pi^{2}}(\ln r)^{2}  \text{ in } {\Omega}_{4}\cap\mathcal{V}_{0} . $$

Note that in this case, the integer *k*_0_ in the statement of the theorem equals 2.

**Case** ***δ***^***1***^***= 0*** In this case, well-posedness is guaranteed and no singularity is generated by the method at the current iteration *k* = 2. Indeed, the method behaves exactly as in the first iteration and all $e_{o,i}^{2}$ are well defined. Thus, we can introduce $\delta ^{2}\in \mathbb {R}$ such that $e_{o,2}^{2}(0,0)=-e_{o,4}^{2}(0,0)=:\delta ^{2}$. Then we are again facing two possible situations: *δ*^2^≠ 0, in which case well-posedness is lost and a $(\ln r)^{2}$ singularity is generated at the next iteration *k* = 3 (i.e., *k*_0_ = 3), or *δ*^2^ = 0, in which case we still have the same behavior as in the first iteration. If we keep going with the same reasoning, we end up with two possible cases: either there exists some integer *k*_0_ > 1 such that all iterates are uniquely defined and regular up to *k*_0_ and both regularity and well-posedness are lost at *k* = *k*_0_, or *δ*^*k*^ = 0 for all *k* ≥ 1, in which case all iterates are well defined and regular. This last case, which has never been encountered in practice, is not treated here as it seems difficult to study convergence properties in this specific situation. □

#### *Proof 9* (Proof of Theorem 8)

Here, we study how the singularity exhibited in Theorem 7 propagates through the next iterations *k* ≥ *k*_0_. To begin, we assume that the algorithm is capable of finding one of the solutions to a problem for which uniqueness is not guaranteed (typically problem (15)). To simplify notations, we introduce the integer *p* ≥ 0 such that *k* = *k*_0_ + *p*. Then, we claim that at iteration *k*, for each $i\in \mathcal {I}$, there exists a regular function ${v_{i}^{k}}\in H^{1}({\Omega }_{i})$ and real coefficients $\left (\gamma _{i,j,m}^{k}\right )_{j,m}$ such that the local error $e_{o,i}^{k}$ is given by

19 $$ e_{o,i}^{k} = {v_{i}^{k}} + {\sum}_{m=0}^{2p+1}{\sum}_{j=0}^{2p}\gamma_{i,j,m}^{k} (\phi-\phi_{i})^{m}(\ln r)^{j} ,  \text{ if } i\in\mathcal{I}_{W} ,  $$

20 $$ e_{o,i}^{k} = {v_{i}^{k}} + {\sum}_{m=0}^{2p+2}{\sum}_{j=0}^{2p+2}\gamma_{i,j,m}^{k} (\phi-\phi_{i})^{m}(\ln r)^{j} ,  \text{ if } i\in\mathcal{I}_{G} .  $$

In addition, for $i\in \mathcal {I}_{W}$, we have $\gamma _{i,2p,m}^{k}=0$ if *m* > 1, which means that the leading singularity in Ω_*i*_ is of type $(\phi -\phi _{i})(\ln r)^{2p}$. And for $i\in \mathcal {I}_{G}$, we have $\gamma _{i,2p+2,m}^{k}=0$ if *m*≠ 0; thus, the leading singularity in Ω_*i*_ is of type $(\ln r)^{2p+2}$.

To prove this, we proceed by induction and use the results of the four lemmas stated earlier.

**Base case** The case *k* = *k*_0_, or equivalently *p* = 0, has already been seen in the proof of Theorem 7. Indeed, we have shown that the Dirichlet step in Ω_*i*_ ($i\in \mathcal {I}_{W}$) led to a local error with a singular part $w_{i}^{k_{0}}$ that matches the one in (19) for *k* = *k*_0_ (see expression (13) or (14)). In addition, the Neumann step in Ω_*i*_ ($i\in \mathcal {I}_{G}$) led to a local error with a singular part $w_{i}^{k_{0}}+C_{i}^{k_{0}}z_{i}$, which can be replaced by $w_{i}^{k_{0}}+C_{i}^{k_{0}}(\ln r)$ up to some changes in the regular part $v_{i}^{k_{0}}$. It follows from expressions (16) and (18) that this matches the singular part in (20) for *k* = *k*_0_.

**Induction step** Let *k* > *k*_0_, or equivalently *p* > 0, be fixed. Assuming our statement holds for any integer *ℓ* ≤ *k*, let us prove that it still holds for *k* + 1. Given $e_{o,i}^{k}$ for each $i\in \mathcal {I}$, the only way to get information about $e_{o,i}^{k+1}$ is to perform the Dirichlet and Neumann steps of the algorithm.

**Dirichlet step:** In Ω_1_ we solve

21 $$ \left\{ \begin{aligned} -{\Delta} e_{o,1}^{k+1} &= 0  \text{ in } {\Omega}_{1} , \\ e_{o,1}^{k+1} &= 0  \text{ on } {\partial{\Omega}_{1}^{0}} , \\ e_{o,1}^{k+1} &= \theta e_{o,2}^{k}+(1-\theta)e_{o,1}^{k}  \text{ on } {\Gamma}_{12} , \\ e_{o,1}^{k+1} &= \theta e_{o,4}^{k}+(1-\theta)e_{o,1}^{k}  \text{ on } {\Gamma}_{41} . \end{aligned}\right.  $$

As previously, we know from [37, Theorem 4.4.3.3] that if there exists a solution to (21) that is in $L^{2}({\Omega }_{1})$, then it is unique. In order to prove existence, we use the induction hypothesis for $e_{o,2}^{k}$ and $e_{o,4}^{k}$, then we decompose $e_{o,1}^{k+1} = \theta \tilde {w}_{1}^{k+1}+(1-\theta )e_{o,1}^{k}$, where $\tilde {w}_{1}^{k+1}$ is for 1 ≤ *j* ≤ 2*p* + 2 the sum of the solutions to the boundary value problems

22 $$ \left\{ \begin{aligned} -{\Delta} v &= 0  \text{ in } {\Omega}_{1} , \\ v &= 0  \text{ on } {\partial{\Omega}_{1}^{0}} , \end{aligned} \right. \text{ and } \begin{aligned} (a)  & \left\{ \begin{aligned} v &= {v_{2}^{k}}  \text{ on } {\Gamma}_{12} , \\ v &= {v_{4}^{k}}  \text{ on } {\Gamma}_{41} . \end{aligned} \right. \\ (b)_{j}  & \left\{ \begin{aligned} v &= \mu_{2,j}^{k} (\ln r)^{j}  \text{ on } {\Gamma}_{12} , \\ v &= -\mu_{2,j}^{k} (\ln r)^{j}  \text{ on } {\Gamma}_{41} . \end{aligned} \right. \\ (c)_{j}  & \left\{ \begin{aligned} v &= \nu_{2,j}^{k} (\ln r)^{j}  \text{ on } {\Gamma}_{12} , \\ v &= \nu_{2,j}^{k} (\ln r)^{j}  \text{ on } {\Gamma}_{41} , \end{aligned} \right. \end{aligned}  $$

where we have introduced for each *j*

$$ \mu_{2,j}^{k} := {\sum}_{n=0}^{p+1} \gamma_{2,j,2n}^{k} \left( \frac{\pi}{4}\right)^{2n}  \text{ and }  \nu_{2,j}^{k} := -{\sum}_{n=0}^{p} \gamma_{2,j,2n+1}^{k} \left( \frac{\pi}{4}\right)^{2n+1} . $$

Note that we have performed some simplifications using the equality $\gamma _{2,j,m}^{k}=-\gamma _{4,j,m}^{k}$ which holds for all *k*, *j*, *m*, due to the odd symmetry of the problem. Now, we consider each part of (22), and express the general form of its solution. First, since ${v_{2}^{k}}$ and ${v_{4}^{k}}$ are smooth and verify ${v_{2}^{k}}(x,y)=-{v_{4}^{k}}(-x,-y)$ for almost all (*x*,*y*) ∈Ω_2_, we have already seen (in problem (12)) that there exists a solution

$$ \tilde{w}_{a}^{k+1}\in \text{span}\{(\phi-\phi_{1})\}+H^{1}({\Omega}_{1}) $$

to part (*a*), where the coefficient in the linear combination depends on the jump ${v_{2}^{k}}(0,0)$ at the cross-point. Then, for each *j*, we get from Lemma 10 that there exists a solution

$$ \begin{aligned} \tilde{w}_{b_{j}}^{k+1}\in  & \mu_{2,j}^{k}\frac{4}{\pi}(\phi-\phi_{1})(\ln r)^{j} \\  & + \text{span}\left\{(\phi-\phi_{1})^{m}(\ln r)^{q}  \mid  3\leq m \leq j+1, 0\leq q \leq j-2 \right\} + H^{1}({\Omega}_{1}) \end{aligned} $$

to part (*b*)_*j*_. In addition, we also get from Lemma 9 that there exists a solution

$$ \tilde{w}_{c_{j}}^{k+1}\in \nu_{2,j}^{k}(\ln r)^{j} + \text{span}\left\{(\phi-\phi_{1})^{m}(\ln r)^{q}  \mid  2\leq m \leq j, 0\leq q \leq j-2 \right\} + H^{1}({\Omega}_{1}) $$

to part (*c*)_*j*_. Summing up all these contributions over the values of *j*, we deduce that there exists a function $\tilde {v}_{1}^{k+1}\in H^{1}({\Omega }_{1})$ and coefficients $\left (\tilde {\gamma }^{k+1}_{1,j,m}\right )_{j,m}$ such that

$$ \tilde{w}_{1}^{k+1} = \tilde{v}_{1}^{k+1} + {\sum}_{m=0}^{2p+3}{\sum}_{j=0}^{2p+2}\tilde{\gamma}_{1,j,m}^{k+1} (\phi-\phi_{i})^{m}(\ln r)^{j} $$

solves (22) in $L^{2}({\Omega }_{1})$, with $\tilde {\gamma }_{1,2p+2,m}^{k+1}=0$ for *m* > 1. Finally, defining $e_{o,1}^{k+1} := \theta \tilde {w}_{1}^{k+1}+(1-\theta )e_{o,1}^{k}$, we obtain a solution to (21) that is in $L^{2}({\Omega }_{1})$, which also proves that it is unique in $L^{2}({\Omega }_{1})$. Moreover, using the previous decomposition for $\tilde {w}_{1}^{k+1}$ and the induction hypothesis for $e_{o,1}^{k}$, we get that there exists a function $v_{1}^{k+1}:=\theta \tilde {v}_{1}^{k+1}+(1-\theta ){v_{1}^{k}}\in H^{1}({\Omega }_{1})$ and coefficients $\left (\gamma ^{k+1}_{1,j,m}\right )_{j,m}$ such that

$$ e_{o,1}^{k+1} = v_{1}^{k+1} + {\sum}_{m=0}^{2p+3}{\sum}_{j=0}^{2p+2}\gamma_{1,j,m}^{k+1} (\phi-\phi_{1})^{m}(\ln r)^{j} , $$

with $\gamma _{1,2p+2,m}^{k+1}=\theta \tilde {\gamma }_{1,2p+2,m}^{k+1}=0$ for *m* > 1. Obviously, the same result (existence, uniqueness, and decomposition) holds for $e_{o,3}^{k+1}$ in Ω_3_ due to the odd symmetry property of the problem.

**Neumann step:** In Ω_2_ we solve

23 $$ \left\{ \begin{aligned} -{\Delta} e_{o,2}^{k+1} &= 0  \text{ in } {\Omega}_{2} , \\ e_{o,2}^{k+1} &= 0  \text{ on } {\partial{\Omega}_{2}^{0}} , \\ \partial_{x} e_{o,2}^{k+1} &= \partial_{x} e_{o,1}^{k+1}  \text{ on } {\Gamma}_{12} , \\ \partial_{y} e_{o,2}^{k+1} &= \partial_{y} e_{o,3}^{k+1}  \text{ on } {\Gamma}_{23} . \end{aligned}\right.  $$

We have already seen (again from [37, Theorem 4.4.3.3]) that, if there exists a solution to (23) that is in $L^{2}({\Omega }_{2})$, then the set of all solutions is an affine subspace of dimension 1. As in the Dirichlet step, in order to prove existence, we decompose $e_{o,2}^{k+1}$ using the decompositions obtained for $e_{o,1}^{k+1}$ and $e_{o,3}^{k+1}$. More specifically, we consider for 1 ≤ *j* ≤ 2*p* + 2 the sum of the solutions to the boundary value problems

24 $$ \left\{ \begin{aligned} -{\Delta} v &= 0  \text{ in } {\Omega}_{2} , \\ v &= 0  \text{ on } {\partial{\Omega}_{2}^{0}} , \end{aligned} \right. \text{ and } \begin{aligned} (a)  & \left\{ \begin{aligned} \partial_{\phi} v &= \partial_{\phi} v_{1}^{k+1}  \text{ on } {\Gamma}_{12} , \\ \partial_{\phi} v &= \partial_{\phi} v_{3}^{k+1}  \text{ on } {\Gamma}_{23} . \end{aligned} \right. \\ (b)_{j}  & \left\{ \begin{aligned} \partial_{\phi} v &= \mu_{1,j}^{k+1} (\ln r)^{j}  \text{ on } {\Gamma}_{12} , \\ \partial_{\phi} v &= -\mu_{1,j}^{k+1} (\ln r)^{j}  \text{ on } {\Gamma}_{23} . \end{aligned} \right. \\ (c)_{j}  & \left\{ \begin{aligned} \partial_{\phi} v &= \nu_{1,j}^{k+1} (\ln r)^{j}  \text{ on } {\Gamma}_{12} , \\ \partial_{\phi} v &= \nu_{1,j}^{k+1} (\ln r)^{j}  \text{ on } {\Gamma}_{23} , \end{aligned} \right. \end{aligned}  $$

where we have introduced for each *j*

$$ \mu_{1,j}^{k+1} := {\sum}_{n=0}^{p+1} \gamma_{1,j,2n+1}^{k+1} (2n+1)\left( \frac{\pi}{4}\right)^{2n}  \text{ and }  \nu_{1,j}^{k+1} := {\sum}_{n=0}^{p} \gamma_{1,j,2n+2}^{k+1}(2n+2) \left( \frac{\pi}{4}\right)^{2n+1} . $$

This time we have used the equality $\gamma _{1,j,m}^{k+1}=-\gamma _{3,j,m}^{k+1}$ to simplify the formulations. Let us now study separately each part of (24), and express the general form of its solution. To begin with, due to the regularities of $v_{1}^{k+1}$ and $v_{3}^{k+1}$, we know from standard results that there exists a (unique) solution

$$ \bar{w}_{a}^{k+1}\in H^{1}({\Omega}_{2}) $$

to part (*a*). Then, for each *j*, we get from Lemma 12 that there exists a solution

$$ \begin{aligned} \bar{w}_{b_{j}}^{k+1} \in  & \mu_{1,j}^{k+1}\frac{4}{\pi}\left( \frac{1}{2}(\phi-\phi_{2})^{2}(\ln r)^{j} -\frac{1}{(j+1)(j+2)}(\ln r)^{j+2} \right) \\  & + \text{span}\left\{(\phi-\phi_{2})^{m}(\ln r)^{q}  \mid  4\leq m \leq j+2, 0\leq q \leq j-2 \right\} + H^{1}({\Omega}_{2}) \end{aligned} $$

to part (*b*)_*j*_. Finally, we get from Lemma 10 that there exists a solution

$$ \begin{aligned} \bar{w}_{c_{j}}^{k+1} \in  & \nu_{1,j}^{k+1}(\phi-\phi_{2})(\ln r)^{j} \\  & + \text{span}\left\{(\phi-\phi_{2})^{m}(\ln r)^{q}  \mid  3\leq m \leq j+1, 0\leq q \leq j-2 \right\} + H^{1}({\Omega}_{2}) \end{aligned} $$

to part (*c*)_*j*_. Combining these results, we end up with a function

$$ v_{2}^{k+1} + {\sum}_{m=0}^{2p+4}{\sum}_{j=0}^{2p+4}\gamma_{2,j,m}^{k+1} (\phi-\phi_{2})^{m}(\ln r)^{j} $$

that solves (24) in $L^{2}({\Omega }_{2})$, where $v_{2}^{k+1}\in H^{1}({\Omega }_{2})$ and the coefficients $\left (\gamma _{2,j,m}^{k+1} \right )_{j,m}$ satisfy $\gamma _{2,2p+4,m}^{k+1}=0$ if *m*≠ 0. This means that every solution $e_{o,2}^{k+1}$ to (24) is given by the general expression

$$ e_{o,2}^{k+1} = v_{2}^{k+1} + {\sum}_{m=0}^{2p+4}{\sum}_{j=0}^{2p+4}\gamma_{2,j,m}^{k+1} (\phi-\phi_{2})^{m}(\ln r)^{j} + C_{2}^{k+1} z_{2} , $$

with $C_{2}^{k+1}\in \mathbb {R}$. Since the singularity in *z*_2_ is of type $\ln r$, it follows that every solution can be written as

$$ e_{o,2}^{k+1} = v_{2}^{k+1} + {\sum}_{m=0}^{2p+4}{\sum}_{j=0}^{2p+4}\gamma_{2,j,m}^{k+1} (\phi-\phi_{2})^{m}(\ln r)^{j} , $$

up to some modification of the regular part $v_{2}^{k+1}$ and the coefficient $\gamma _{2,1,0}^{k+1}$. Again, the same result can be deduced for $e_{o,4}^{k+1}$ in Ω_4_.

This ends the proof of our claim about the expression of the local errors $e_{o,i}^{k}$ (see (19) and (20)). The type of singularity generated by the domain decomposition method at iteration *k* is directly given by those expressions, so the proof of the result is complete. □

#### *Remark 2*

Following the same computations as in the previous proof while taking care of the coefficient in front of the leading singularity at each step enables us to end up with the following approximations of the errors in subdomains ${\Omega }_{1}, \dots , {\Omega }_{4}$ at iteration *k* = *k*_0_ + *p*

25 $$ \begin{aligned} e_{o,i}^{k} \simeq& \pm \theta\delta^{1}\frac{4}{\pi}(\phi-\phi_{1})\frac{1}{(2p)!}\left( \theta\left( \frac{4}{\pi} \ln r\right)^{2} \right)^{p},  & \text{ for } i\in\mathcal{I}_{W} , \\ e_{o,i}^{k} \simeq& \pm\delta^{1}\frac{1}{(2p+2)!}\left( \theta\left( \frac{4}{\pi} \ln r\right)^{2} \right)^{p+1},  & \text{ for } i\in\mathcal{I}_{G} , \end{aligned}  $$

where the correct sign is + for *i* ∈{1,2} and − for *i* ∈{3,4}.

The results given by Theorem 6, Theorem 7, and Theorem 8 can be easily extended to the Laplace problem with Robin boundary conditions. In this case, no compatibility condition is required, we only impose that the initial guess *λ*^0^ satisfies the regularity assumption that $\lambda ^{0} \in C^{0}({\Gamma })\cap H^{\frac {3}{2}}({\Gamma }_{ij})$ for all (*i*,*j*) such that Γ_*ij*_≠*∅*.

#### **Theorem 13**

Taking ${\lambda ^{0}_{e}}$ as the initial guess for the Dirichlet-Neumann method applied to the even symmetric part of problem (2) produces a sequence $\{{u_{e}^{k}}\}_{k}$ that converges geometrically to the solution *u*_*e*_ with respect to the *L*^2^-norm and the broken *H*^1^-norm. Moreover, the convergence factor is given by ∣1 − 2*𝜃*∣, which also proves that this method becomes a direct solver for the specific choice $\theta =\frac {1}{2}$.

#### **Theorem 14**

The Dirichlet-Neumann method applied to the odd symmetric part of problem (2) is not well-posed. More specifically, taking ${\lambda ^{0}_{o}}$ as the initial guess, there exists an integer *k*_0_ > 0 such that the solution to the problem obtained at the *k*_0_-th iteration is not unique. In addition, all possible solutions $u_{o}^{k_{0}}$ are singular at the cross-point, with a leading singularity of type $(\ln r)^{2}$.

#### **Theorem 15**

If we let the Dirichlet-Neumann method go beyond the ill-posed iteration *k*_0_ from Theorem 14, we end up with a sequence $\{{u_{o}^{k}}\}_{k\geq k_{0}}$ of non-unique iterates. Moreover, for each *k* ≥ *k*_0_, all possible ${u_{o}^{k}}$ are singular at the cross-point, with a leading singularity of type $(\ln r)^{2(k-k_{0})+2}$.

#### *Proof 10*

The proofs can be obtained by following the same steps as in the proofs of Theorem 6, Theorem 7 and Theorem 8. □

#### *Remark 3*

Note that the formulas given by the asymptotic analysis near the origin remain valid in the Robin case. Indeed, as this study has been conducted in the neighborhood of (0,0), what happens “far away” from this point (e.g., on the boundary *∂*Ω) has no influence on the results.

## Numerical experiments

We now illustrate the theoretical results obtained in the previous section. The square domain Ω is discretized using a regular grid of size *h*, and our numerical method is based on a standard five-point finite difference scheme. In problems with mixed boundary conditions (Dirichlet and Robin), the Dirichlet boundary condition is enforced weakly using a penalty parameter *ε* of order 10^− 10^. Unless otherwise stated, the mesh size will be set to *h* = 2 ⋅ 10^− 2^ in all experiments. In addition, each convergence analysis is performed taking *u*_*ex*_ (exact solution) as the discrete solution obtained when solving on the whole domain Ω with a direct solver.

### Example 1

**Fig. 3:**
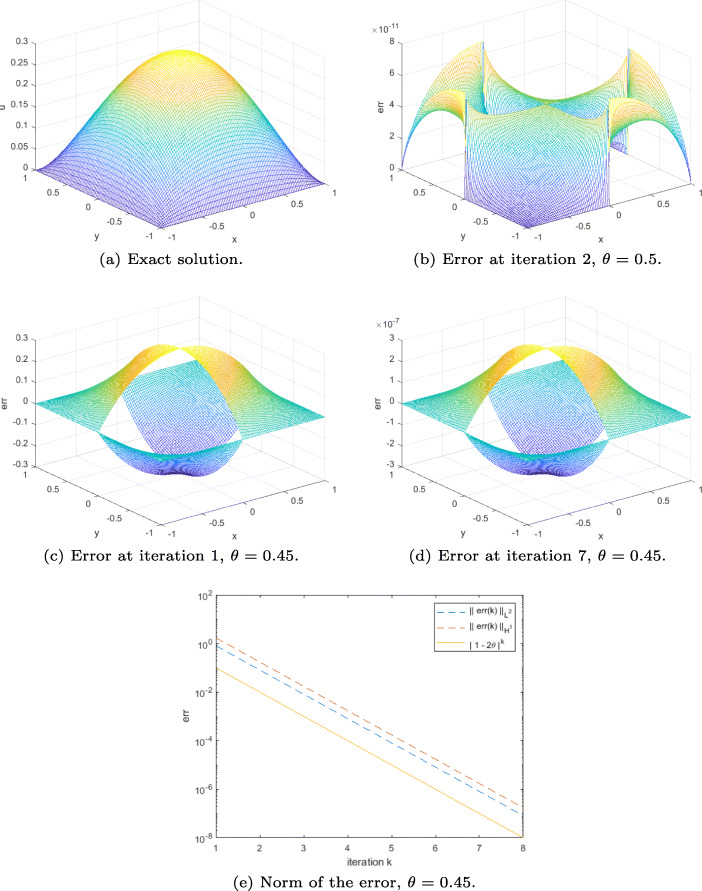
Results for the DN method applied to (6) (Example 1)

In order to illustrate the result of Theorem 6, i.e., convergence for the even symmetric part, we take the source term *f* = *f*_*e*_ = 1 in Ω, and set the Dirichlet boundary condition to *g* = *g*_*e*_ = 0 on *∂*Ω. A simple initial guess compatible with the Dirichlet boundary condition is in this case $\lambda ^{0}={\lambda _{e}^{0}}=0$ on Γ. The results are displayed in Fig. 3.

As expected, when $\theta =\frac {1}{2}$, the DN method becomes a direct solver; thus, the error at iteration 2 is “zero” (here it cannot be smaller than the order of magnitude of *ε*) (see Fig. 3b). For $\theta \neq \frac {1}{2}$, we see from Fig. 3c and d that the error on Ω is multiplied by a constant from one iteration to the next. Moreover, the plot of the *L*^2^ and broken *H*^1^ norms of the error in Fig. 3e confirms that the DN method converges geometrically in this case. We also see that, as predicted by the proof of Theorem 6, at each iteration, the error remains continuous at the cross-point from Ω_1_ to Ω_3_, and from Ω_2_ to Ω_4_, and it has the expected symmetry property.

### Example 2

**Fig. 4:**
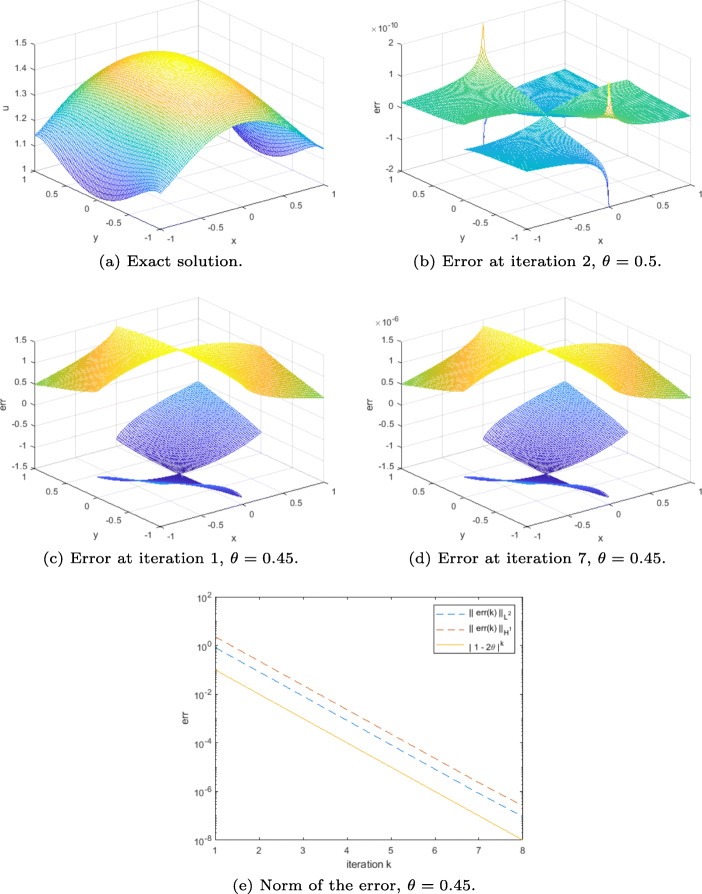
Results for the DN method applied to the even symmetric part of (2) (Example 2)

We illustrate now the result of Theorem 13 for the even symmetric part with Robin conditions: the source term is *f* = *f*_*e*_ = 1 in Ω, and the Robin boundary condition is defined by *p* = *p*_*e*_ = 1 and *g* = *g*_*e*_ on *∂*Ω, where *g*_*e*_ is such that *g*_*e*_ = 1 on *∂*Ω_*b*_ ∪ *∂*Ω_*t*_ (bottom and top sides) and *g*_*e*_ = *y*^2^ on *∂*Ω_*l*_ ∪ *∂*Ω_*r*_ (left and right sides). The same initial guess $\lambda ^{0}={\lambda _{e}^{0}}=0$ on Γ is considered. As shown in Fig. 4, we observe the same convergence properties as for the Dirichlet problem (Example 1). Especially, the DN method becomes a direct solver when *𝜃* is set to $\frac {1}{2}$ (see Fig. 4b), and for other choices of *𝜃*, it converges geometrically (see Fig. 4e), with the expected common ratio (1 − 2*𝜃*).

### Example 3

**Fig. 5:**
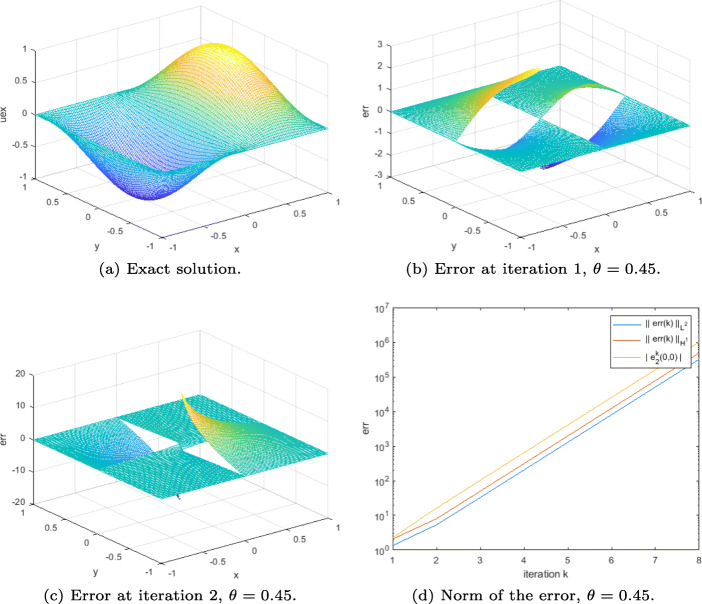
Results for the DN method applied to (7) (Example 3)

Finally, we give an illustration of the problematic case described in Theorem 7 and Theorem 8. We consider a Dirichlet problem with the odd symmetric data: $f=f_{o}=\frac {5\pi ^{2}}{4}\sin \limits (\pi x)\cos \limits (\frac {\pi }{2}y)$ in Ω and *g* = *g*_*o*_ = 0 on *∂*Ω. As in the previous examples, the initial guess is set to $\lambda ^{0}={\lambda ^{0}_{o}}=0$. The results displayed in Fig. 5 show that, as expected, the DN method applied to this odd symmetric problem does not converge. More specifically, we see that after the first iteration (see Fig. 5b), continuity across the cross-point from Ω_2_ to Ω_4_ is already lost. This jump (referred to as *δ*^1^≠ 0 in the proof of Theorem 7) generates a “singularity” at the next iteration (see Fig. 5c). This “singularity” keeps propagating in the following iterations so that the method diverges, as predicted by Theorem 8. Moreover, the graph in Fig. 5d reveals a geometric (divergent) behavior of the errors in *L*^2^ and broken *H*^1^ norms.

#### *Remark 4*

Since we use a standard finite difference scheme, it is not possible to enforce a discontinuous Dirichlet boundary condition. In practice, when two Dirichlet boundary values do not match at a corner, the average is computed and imposed at this corner. Thus, every numerical solution in Ω_*i*_ necessarily belongs to a finite dimensional subspace of $C^{0}(\overline {{\Omega }_{i}})$, which explains the quotation marks for singularity in the previous paragraph. More generally, solving this problem using standard discretization methods (such as the finite difference method or the finite element method) involves a regularization step, namely the projection of the discontinuous boundary condition onto some finite dimensional subspace of $C^{0}(\overline {{\Omega }_{i}})$.

**Fig. 6:**
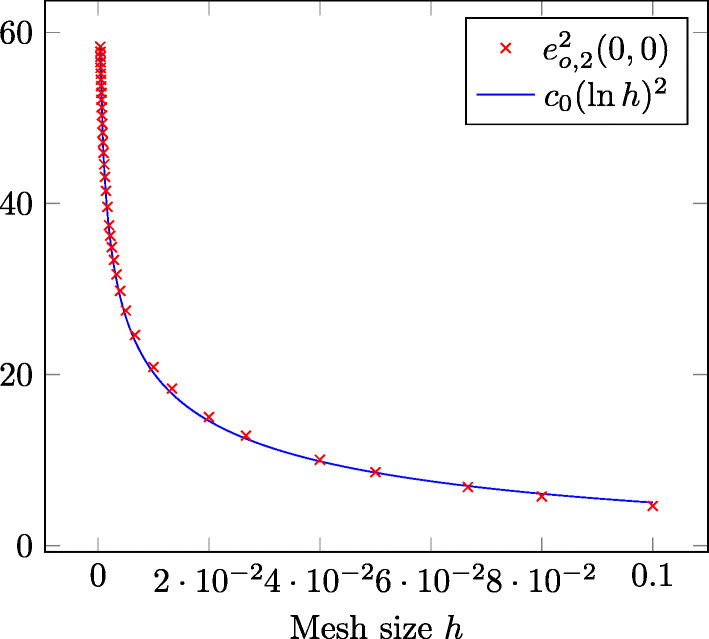
Error at the cross-point at iteration 2 with respect to *h*, for the DN method applied to the odd symmetric problem described in Example 3, with *𝜃* = 0.45

Given our choice of projection (computing the average), the boundary data are regularized in the disk *D*_*h*_ of radius *h* centered at the origin. Outside this disk, they are not modified. Consequently, one may argue that, in each subdomain Ω_*i*_, the local numerical solution should be “not too far” from the local real solution in Ω_*i*_ ∖ *D*_*h*_. In order to verify this in the numerical results, we have plotted (red marks) the value of the error at the cross-point in subdomain Ω_2_ with respect to the mesh size *h* (see Fig. 6). We see that these points follow a curve of $(\ln h)^{2}$ type. An interpretation of that result is that the discretization process (which acts as a regularization here) turns the singularity of type $(\ln r)^{2}$ from Theorem 7 (see formula (17)) into a pseudo “singularity” of type $(\ln h)^{2}$.

Now, we would like to analyze how the “singularity” propagates through the numerical iterates. In other words, given the “singularity” of type $(\ln h)^{2}$ obtained at iteration *k* = *k*_0_, do we observe a “singularity” of type $(\ln h)^{2p+2}$ at the following iterations *k* = *k*_0_ + *p* with *p* > 0, as predicted by Theorem 8 ? To answer this, let us first note that the error at the cross-point $\mid e_{o,2}^{k}(0,0)\mid $ seems to grow geometrically with respect to *k* (see Fig. 5d). Thus, for each *h*, we are able to compute constants *α*_2_, *β*_2_ such that, for *k* ≥ 2,

$$ \ln \mid e_{o,2}^{k}(0,0) \mid  \simeq  \alpha_{2} k + \beta_{2} . $$

**Fig. 7:**
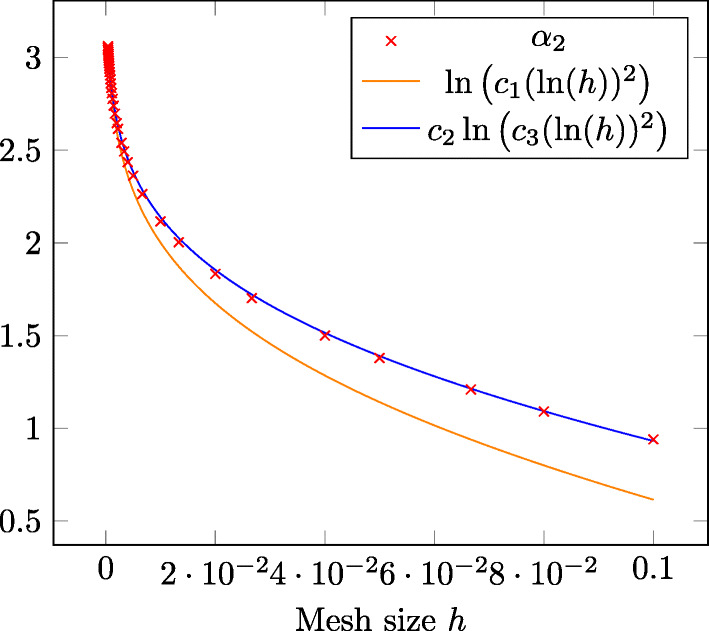
Slope of the curve $k\mapsto \ln \mid e_{o,2}^{k}(0,0)\mid $ with respect to *h*, for the DN method applied to the odd symmetric problem described in Example 3, with *𝜃* = 0.45

In addition, computing the logarithm of formula (25) (with *i* = 2), we get in the neighborhood of the cross-point

$$ \ln \mid e_{o,2}^{k} \mid  \simeq  \ln \left( \theta\left( \frac{4}{\pi} \ln r\right)^{2}\right) k + \tilde{\beta}_{2} , $$

where $\tilde {\beta }_{2}$ depends on *𝜃* and *k* (only logarithmically). In Fig. 7, we have plotted the value computed for the coefficient *α*_2_ as a function of the mesh size *h*, and tried to make it fit with a curve of type $\ln (c_{1}(\ln h)^{2})$ (drawn in orange). As shown in the figure, this fitting was not successful and it appears that the appropriate fitting is a curve of type $c_{2}\ln (c_{3}(\ln h)^{2})$ (drawn in blue), with a constant $c_{2}\simeq 0.87$. This suggests that, in the numerical experiments, the “singularity” at iteration *k* = *k*_0_ + *p* is of type $(\ln h)^{1.74(p+1)}$ rather than $(\ln h)^{2(p+1)}$. One possible explanation for this is the regularizing effect of the discretization, which may slow down the propagation of the “singularity.”

## Conclusion

We presented a complete analysis of the Dirichlet-Neumann method at the continuous level in a specific configuration involving one cross-point. Based on an even/odd symmetric decomposition of the data, we proved that the even symmetric part of the iterates converges geometrically to the right solution, while the boundary value problems associated to the odd symmetric part are not well-posed, which generates singular iterates. We also exhibited the type of singularity generated, and showed how this singularity propagates through the iterations. Finally, we studied the impact of our theoretical findings on numerical experiments.

A natural extension of this work would be to conduct a similar analysis for the Neumann-Neumann method, which is also known to pose problems in configurations with cross-points (see [41]). Another direction of future work, which will be the subject of the second part of this paper, is to build a modified (and convergent) Dirichlet-Neumann method taking advantage of this even/odd symmetric decomposition of the data.

## Footnotes

1 The same result was already proved in [2], in the case of a symmetric decomposition with two subdomains without any restriction on the even/odd symmetric nature of the data.

## References

[1] Schwarz HA (1870). Über einen Grenzübergang durch alternierendes Verfahren. Vierteljahrsschrift der Naturforschenden Gesellschaft in Zürich.

[2] Bjørstad PE, Widlund OB (1986). Iterative methods for the solution of elliptic problems on regions partitioned into substructures. SIAM J. Numer. Anal..

[3] Bourgat, J.-F., Glowinski, R., Le Tallec, P., Vidrascu, M., Glowinski, R., Périaux, J., Widlund, O: Variational formulation and algorithm for trace operator in domain decomposition calculations. In: Chan, T. (ed.) Domain decomposition methods, pp 3–16, SIAM (1989)

[4] Farhat C, Roux F-X (1991). A method of Finite Element Tearing and Interconnecting and its parallel solution algorithm. Int. J. Numer. Meth. Engrg..

[5] Toselli, A., Widlund, O.: Domain Decomposition Methods - Algorithms and Theory. Springer Series in Computational Mathematics, vol. 34. Springer (2004)

[6] Hackbusch W (1976). Ein iteratives Verfahren zur schnellen Auflösung elliptischer randwertprobleme, rep. 76-12 Technical Report 76–12.

[7] Brandt A (1977). Multi-level adaptive solutions to boundary value problems. Math. Comp..

[8] Quarteroni, A., Valli, A.: Domain Decomposition Methods for Partial Differential Equations. Oxford University Press (1999)

[9] Lions, P.-L.: On the Schwarz alternating method. I. In: Glowinski, R., Golub, G.H., Meurant, G.A. , Périaux, J. (eds.) First International Symposium on Domain Decomposition Methods for Partial Differential Equations, pp. 1–42. SIAM (1988)

[10] Efstathiou E, Gander MJ (2003). Why Restricted Additive Schwarz converges faster than Additive Schwarz. BIT Numer. Math..

[11] Gander MJ (2008). Schwarz methods over the course of time. Electron. Trans. Numer. Anal.

[12] Després B (1991). Méthodes de décomposition de domaine pour les problèmes de propagation d’ondes en régimes harmoniques.

[13] Lions, P.-L.: On the Schwarz alternating method. III:, a variant for nonoverlapping subdomains. In: Chan, T.F., Glowinski, R., Périaux, J., Widlund, O. (eds.) Third International Symposium on Domain Decomposition Methods for Partial Differential Equations, Held in Houston, Texas, March 20-22, 1989. SIAM (1990)

[14] Boubendir, Y., Bendali, A.: Dealing with cross-points in a non-overlapping domain decomposition solution of the Helmholtz equation. In: Mathematical and Numerical Aspects of Wave Propagation WAVES 2003, pp 319–324. Springer, Skylä (2003)

[15] Gander MJ, Kwok F (2012). Best Robin parameters for optimized Schwarz methods at cross points. SIAM J. Sci. Comput..

[16] Loisel S (2013). Condition number estimates for the nonoverlapping optimized Schwarz method and the 2-Lagrange multiplier method for general domains and cross points. SIAM J. Numer. Anal..

[17] Gander, M.J., Kwok, F.: On the applicability of Lions’ energy estimates in the analysis of discrete optimized Schwarz methods with cross points. In: Domain Decomposition Methods in Science and Engineering XX, pp. 475–483. Springer (2013)

[18] Gander MJ, Santugini-Repiquet K (2016). Cross-points in domain decomposition methods with a finite element discretization. ETNA.

[19] Bamberger A, Joly P, Roberts JE (1990). Second-order absorbing boundary conditions for the wave equation: a solution for the corner problem. SIAM J. Num. Anal..

[20] Nataf F (1998). A Schwarz additive method with high order interface conditions and nonoverlapping subdomains. ESAIM: Mathematical Modelling and Numerical Analysis-Modélisation Mathématique et Analyse Numérique.

[21] Modave A, Royer A, Antoine X, Geuzaine C (2020). A non-overlapping domain decomposition method with high-order transmission conditions and cross-point treatment for Helmholtz problems. Comput. Methods Appl. Mech. Eng..

[22] Després, B., Nicolopoulos, A., Thierry, B.: Corners and stable optimized domain decomposition methods for the Helmholtz problem hal-02612368 (2020)

[23] Gander, M.J., Halpern, L.: A simple finite difference discretization for Ventcell transmission conditions at cross points. In: Domain Decomposition Methods in Science and Engineering XXVI. Springer (2021)

[24] Chaouqui, F., Gander, M.J., Santugini-Repiquet, K.: A local coarse space correction leading to a well-posed continuous neumann-neumann method in the presence of cross points. In: International Conference on Domain Decomposition Methods, pp. 83–91. Springer (2018)

[25] Claeys, X., Parolin, E.: Robust treatment of cross points in optimized S,chwarz methods. arXiv:2003.06657 (2020)

[26] Gander, M.J., Kwok, F.: Optimal interface conditions for an arbitrary decomposition into subdomains. In: Domain Decomposition Methods in Science and Engineering XIX, pp. 101–108. Springer (2011)

[27] Domain Decomposition Methods in Science and Engineering XXVI. Springer, Hong-Kong (2021)

[28] De Roeck, Y. -H., Le Tallec, P.: Analysis and test of a local domain-decomposition preconditioner. In: Domain Decomposition Methods in Science and Engineering IV. Springer (1990)

[29] Maier I, Haasdonk B (2014). A dirichlet–neumann reduced basis method for homogeneous domain decomposition problems. Appl. Numer. Math..

[30] Song B, Jiang Y-L, Wang X (2021). Analysis of two new parareal algorithms based on the dirichlet-neumann/neumann-neumann waveform relaxation method for the heat equation. Numer. Algor..

[31] Chaouqui F, Ciaramella G, Gander MJ, Vanzan T (2018). On the scalability of classical one-level domain-decomposition methods. Vietnam J. Math..

[32] Gander MJ, Kwok F, Mandal BC (2021). Dirichlet–neumann waveform relaxation methods for parabolic and hyperbolic problems in multiple subdomains. BIT Numer. Math..

[33] Widlund, O., Dryja, M., Proskurowski, W.: A method of domain decomposition with crosspoints for elliptic finite element problems. In: Proceedings of the International Symposium on Optimal Algorithms. Blagovegrad, Bulgaria, April 21-25, 1986, pp. 97–111. Bulgarian Academy of Sciences, Sofia (1986)

[34] Dryja, M.: A method of domain decomposition for three-dimensional finite element elliptic problems. In: First International Symposium on Domain Decomposition Methods for Partial Differential Equations, pp 43–61. SIAM, Philadelphia (1988)

[35] Parolin É (2020). Non-overlapping Domain Decomposition Methods with Non-local Transmissionoperators for Harmonic Wave Propagation Problems.

[36] Dauge, M.: Elliptic Boundary Value Problems on Corner Domains: Smoothness and Asymptotics of Solutions vol. 1341. Springer (2006)

[37] Grisvard, P.: Elliptic Problems in Nonsmooth Domains. SIAM (2011)

[38] Dauge, M.: Regularity and singularities in polyhedral domains. Available on: https://perso.univ-rennes1.fr/monique.dauge/publis/Talk_Karlsruhe08.pdf (2008). Accessed 16 Sept 2021

[39] Demlow, A.: Lecutre notes: Singular solutions to pde and their finite element approximations. Available on: https://www.math.tamu.edu/~demlow/Courses/16_sp/663_sp16/index.html (2016). Accessed 1 Aug 2021

[40] Brézis, H.: Functional Analysis, Sobolev Spaces and Partial Differential Equations vol. 2. Springer (2011)

[41] Chaouqui, F., Gander, M.J., Santugini-Repiquet, K.: A coarse space to remove the logarithmic dependency in neumann-neumann methods. In: International Conference on Domain Decomposition Methods, pp 159–167. Springer (2017)

